# Angiotensin signaling is essential for stress erythropoiesis but causes retention of dysfunctional mitochondria in RBCs

**DOI:** 10.1172/jci.insight.200722

**Published:** 2026-03-12

**Authors:** Parul Rai, Swarnava Roy, Paritha Arumugam, Diamantis G. Konstantinidis, Sithara Raju Ponny, Marthe-Sandrine Eiymo Mwa Mpollo, Archana Shrestha, Theodosia A. Kalfa, Punam Malik

**Affiliations:** 1 Division of Hematology,; 2 Cancer and Blood Diseases Institute,; 3 Division of Experimental Hematology and Cancer Biology, and; 4 Genomic Sequencing Facility, Cincinnati Children’s Hospital Medical Center, and; 5 Department of Pediatrics, University of Cincinnati College of Medicine, Cincinnati, Ohio, USA.

**Keywords:** Cell biology, Hematology, Cell stress, Mitochondria

## Abstract

We previously reported that excessive angiotensin-II→AT receptor-1 (AT→ATR1) signaling results in sickle cell anemia–associated (SCA-associated) nephropathy. Herein, we showed that hyperangiotensinemia in SCA results from high erythroid cell–generated reactive oxygen species (ROS), which oxidized angiotensinogen (ATGN) and favored its rapid conversion to AT. Increased AT→ATR1 signaling in SCA erythroid cells generated ROS and created a positive feedback loop of ROS→oxidized ATGN→AT→ATR1→ROS, perpetuating the hyperangiotensinemia. ATR1 blocker, losartan, reduced erythrocyte ROS, oxidized ATGN, and AT levels. The ROS→AT→ATR1→ROS loop was driven by sickle erythropoiesis, as it was reproduced when WT mice were transplanted with SCA hematopoiesis. Using SCA and WT mice with germline- and erythroid-specific ATR1 deficiency, we found that stress erythropoiesis, but not steady-state erythropoiesis, was critically dependent on erythroid AT→ATR1 signaling, which acted in harmony with increased erythropoietin signaling. Furthermore, instead of the canonical AT→ATR1→NADPH-oxidase→ROS signaling in steady-state erythropoiesis, AT→ATR1 signaling in stress erythroid cells increased mitochondrial mass and dysfunctional mitochondria, which thereby increased ROS. SCA mice with erythroid-specific ATR1 deficiency had decreased RBC accumulation of dysfunctional mitochondria and decreased ROS, which reduced SCA-associated nephropathy. Overall, we demonstrate that AT→ATR1 signaling was essential for stress erythropoiesis but led to increased dysfunctional mitochondria retention in mature RBCs, which generated ROS and perpetuated hyperangiotensinemia, resulting in end-organ damage.

## Abstract 2

<strong>We explore the role of angiotensin signaling in stress erythropoiesis, where it sustains erythroid expansion but also promotes retention of dysfunctional mitochondria in erythroid cells</strong>

## Graphical abstract

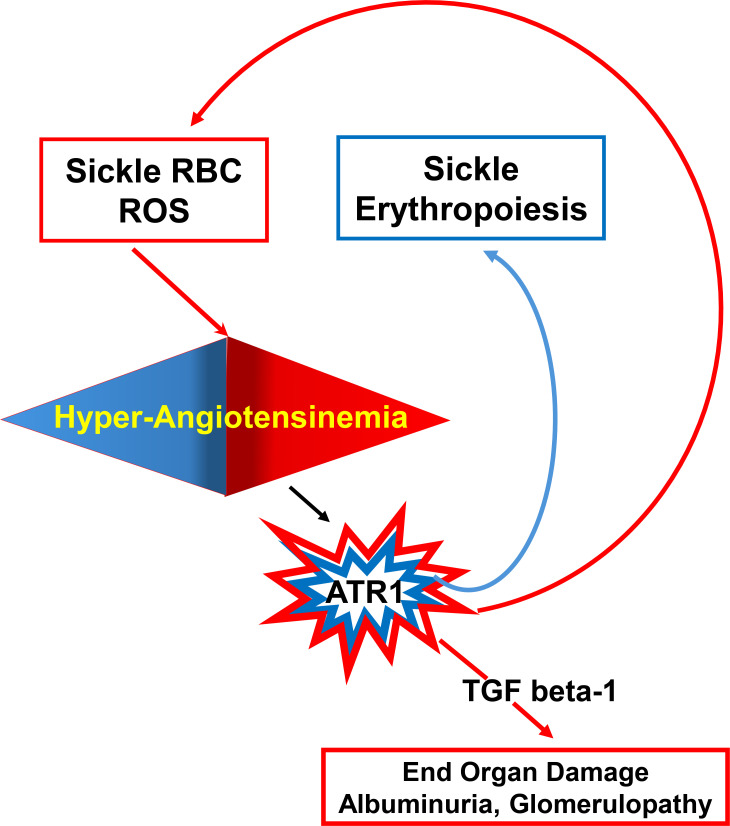

## Introduction

Hyperactivation of the renin-angiotensin signaling (RAS) in sickle cell anemia (SCA) has been shown to promote glomerular injury via the TGF-β1/Smad 2/3 pathway, leading to sickle nephropathy (1, 2). However, the etiology of this hyperangiotensinemia in SCA is unclear, as it occurs early in childhood, in the absence of hyperreninemia or hypertension (2). Reactive oxygen species (ROS) have been shown to modulate the redox state of angiotensinogen (ATGN), the precursor molecule of angiotensin peptides. The oxidized version of ATGN, which is formed in excess from the increased ROS, is converted much more rapidly to angiotensin I and then angiotensin II (AT), the active molecule (3). High oxidative stress is a key feature in the pathophysiology of SCA (4–7) and known to play a role in SCA complications (8–10). Whether the high ROS in SCA contributes to hyperangiotensinemia by increasing oxidized ATGN levels remains unknown.

In SCA, a substantial portion of the sickle erythrocyte ROS is generated enzymatically by engaging the canonical NADPH oxidase (NOX) pathway (11). Furthermore, abnormal retention of mitochondria in RBCs and reticulocytes, accompanied by downregulation of mitophagy inducers, has been observed in patients with SCA and sickle and phlebotomy-stressed mouse models (12–14). These abnormally retained mitochondria in stressed erythrocytes are associated with elevated ROS levels and increased oxygen consumption (12, 13). In nonerythroid cells, AT-induced ROS production has been shown by stimulating NOX activity (15–19) and by induction of mitochondrial dysfunction, indicating a pathophysiological role of NOX-mitochondria cross-talk in ROS production (20–23). Given that the angiotensin II receptor type 1 (ATR1) is expressed on erythroid cells, we sought to investigate the role of AT→ATR1 signaling in mitochondrial retention and ROS production in stressed erythroid cells, including in SCA.

Chronic stimulation of the RAS pathway has also been shown to enhance steady-state erythropoiesis, leading to elevated hematocrit levels (24, 25). This was observed even when erythroid cells were deficient in ATR1 and occurred primarily due to the upregulation of renal erythropoietin (Epo) secretion by AT (24, 26). These studies suggested that AT→ATR1 signaling in erythroid cells did not play a crucial role in steady-state erythropoiesis. However, whether AT→ATR1 signaling plays a role in stress erythropoiesis states, such as SCA, remains to be evaluated.

In this study, we investigate the mechanism underlying the hyperangiotensinemia observed in SCA. We demonstrate that AT→ATR1 signaling is an important driver of ROS production in both normal-stressed erythrocytes and sickle erythrocytes via mitochondria retention. We further show that AT→ATR1 signaling in erythroid cells is essential for sustaining stress erythropoiesis, but this comes at the cost of increased ROS production.

## Results

### Increased ROS in SCA results in hyperangiotensinemia

**Figure 1:**
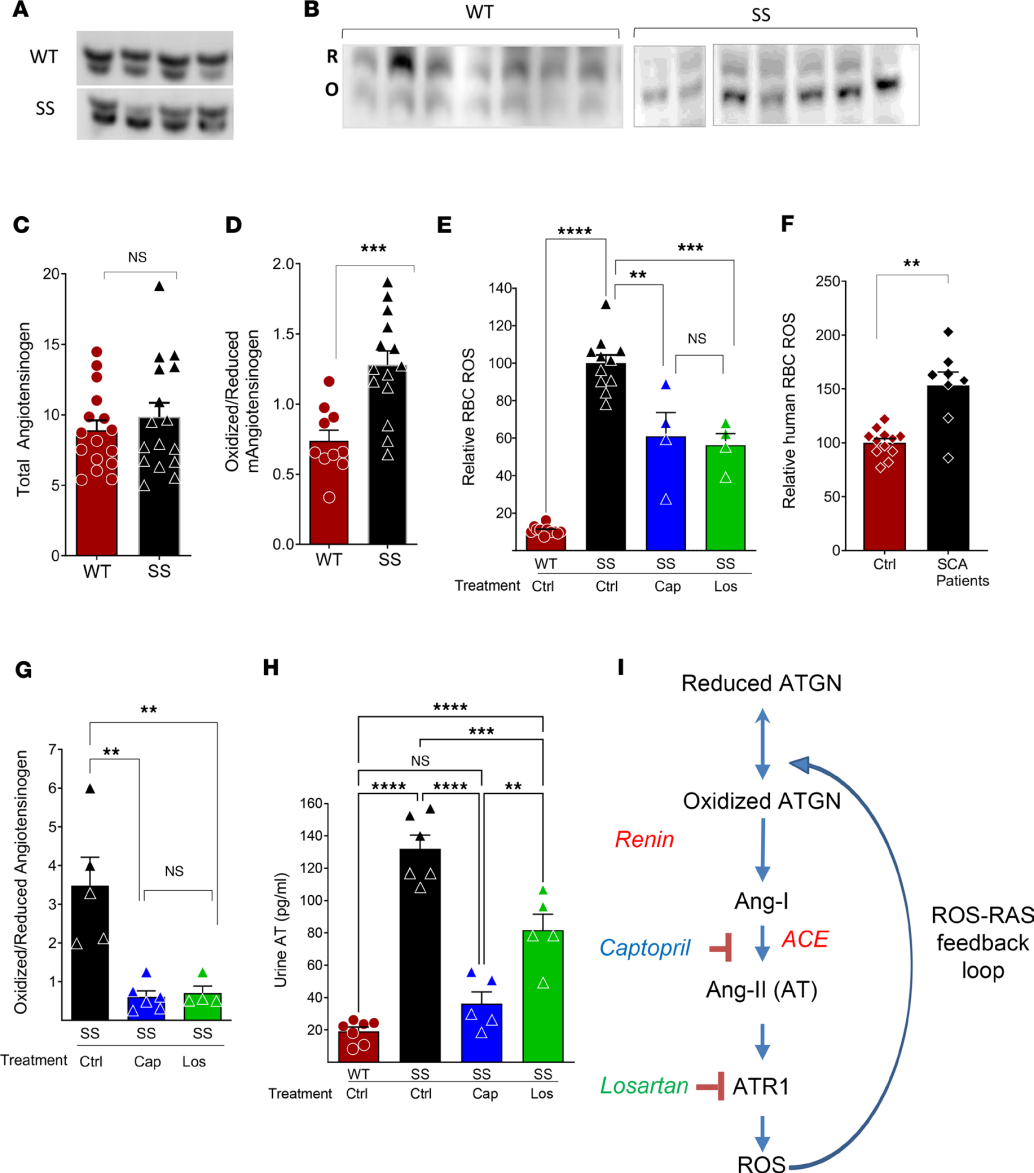
High reactive oxygen species (ROS) in SCA result in the over-activation of the renin-angiotensin system (RAS). (**A** and **B**) Representative Western blot analysis of plasma from WT and Berkeley model (SS) mice for total angiotensinogen and oxidized (O) and reduced (R) angiotensinogen. (**C** and **D**) Quantification of the intensities of total angiotensinogen (*n* = 16 mice each) and oxidized: reduced angiotensinogen (WT *n* = 10 mice, SS *n* = 14 mice). (**E** and **F**) Cumulative data on the mean fluorescence intensities (MFI) of CM-H_2_-DCFDA labeling in RBC showing RBC ROS, with the relative change in SS mean RBC ROS is compared with the WT (or healthy control) mean RBC ROS. (**E**) RBC ROS in SS mice treated with captopril (Cap; blue bar), losartan (Los; green bar), or vehicle (Ctrl; SS black bar and WT red bar) (*n* = 4–11 mice/group). (**F**) RBC ROS in patients with SCA compared with healthy sibling controls (*n* = 9–12 individuals/group). (**G** and **H**) Quantification of the intensity of oxidized: reduced plasma angiotensinogen and urine angiotensin II (AT) levels in SS mice treated with captopril, losartan, and vehicle (*n* = 4–7 mice/group). (**I**) Cartoon of the ROS-RAS feedback loop. Each symbol or lane represents an individual mouse or subject. Statistical analysis in **C**, **D**, and **F** was done using unpaired *t* test, and in **E**, **G**, and **H** was done using 1-way ANOVA. **P < 0.01, ***P < 0.001, ****P < 0.0001.

We evaluated whether the high ROS levels in SCA influence the redox state of ATGN. While the total plasma ATGN levels were similar in WT mice and Berkeley SCA mice (termed SS mice) (Figure 1, A and C), the oxidized ATGN levels were significantly higher in SS mice than in controls (Figure 1, B and D). Similar findings were observed in the plasma samples of patients with SCA compared with their age-matched healthy sibling controls (Supplemental Figure 1, A and B; supplemental material available online with this article; https://doi.org/10.1172/jci.insight.200722DS1). Increased oxidation of ATGN favors its rapid conversion to AT. Hence, higher oxidized ATGN could be the underlying etiology of hyperangiotensinemia reported in mice and humans with SCA (1, 2).

### Blockade of AT→ATR1 signaling lowers sickle RBC ROS and interrupts the ROS-RAS feedback loop

Previous studies examining ROS levels in circulating blood cells have shown higher ROS levels in RBCs of mice and humans with SCA compared with non-SCA controls (7, 27–29). Consistent with prior reports, we found that peripheral blood RBCs from SCA mice (Figure 1E) and patients with SCA (Figure 1F) exhibited significantly higher ROS levels than their respective controls (Figure 1, E and F, and Supplemental Figure 1C).

Our group previously showed that increased NOX signaling in sickle RBC generates ROS (11). The AT→ATR1 pathway has been shown to induce ROS production in nonerythroid cells by engaging NOX (17–19). ATR1, encoded by the *AGTR1* gene, is the primary AT receptor and has been reported to be present on human erythroid cells (26). To evaluate the role of AT→ATR1 signaling in ROS production in sickle RBC, we first pharmacologically interrupted the AT signaling pathway in vivo in SS mice by administering either captopril (an angiotensin-converting enzyme inhibitor [ACE-I] that blocks AT production) or losartan (an ATR1 blocker that would block AT signaling despite high circulating AT levels). Both captopril and losartan significantly reduced ROS in sickle RBCs to similar levels (Figure 1E), consistent with the fact that lowering AT levels reduces ROS, conceivably by reducing AT-ATR1→NOX signaling downstream of AT signaling. The lowering of ROS with both drugs was associated with reduced oxidized ATGN and reduced AT levels (Figure 1, G–I, and Supplemental Figure 1D). The reduction in oxidized ATGN was similar with AT or ATR1 blockade (Figure 1G). Hence, AT signaling predominantly influences ROS production via ATR1, which we studied further.

Mice on losartan, which blocks ATR1 but not AT production, also had reduced AT levels (Figure H), likely due to decreased RBC ROS and reduced oxidized ATGN levels (Figure 1, E and G, and Supplemental Figure 1D). Hence, it appeared that AT→ATR1 signaling in RBC contributes to ROS production, which mediates higher oxidized ATGN and hyperangiotensinemia in SCA. These findings were confirmed in WT mice transplanted with SS bone marrow. The SS chimeric mice (SS→WT) developed higher RBC ROS than their WT chimeras (WT→WT) (Supplemental Figure 1E). When SS chimeric mice were treated with captopril or losartan and compared with untreated SS chimeric mice, the former had significantly lower RBC ROS and AT levels (Supplemental Figure 1, E and F), confirming that the increased RAS-ROS signaling was mediated via sickle hematopoiesis or, more likely, via sickle erythropoiesis. Taken together, these data support our hypothesis that SCA RBC ROS is the driver of increased AT and the RAS-ROS feedback loop in SCA, wherein AT→ATR1 signaling–induced RBC ROS activates RAS, and vice versa (Figure 1I).

### Germline deficiency of AT signaling lowers RBC ROS but results in profound anemia in SCA mice, though not in WT mice

**Figure 2:**
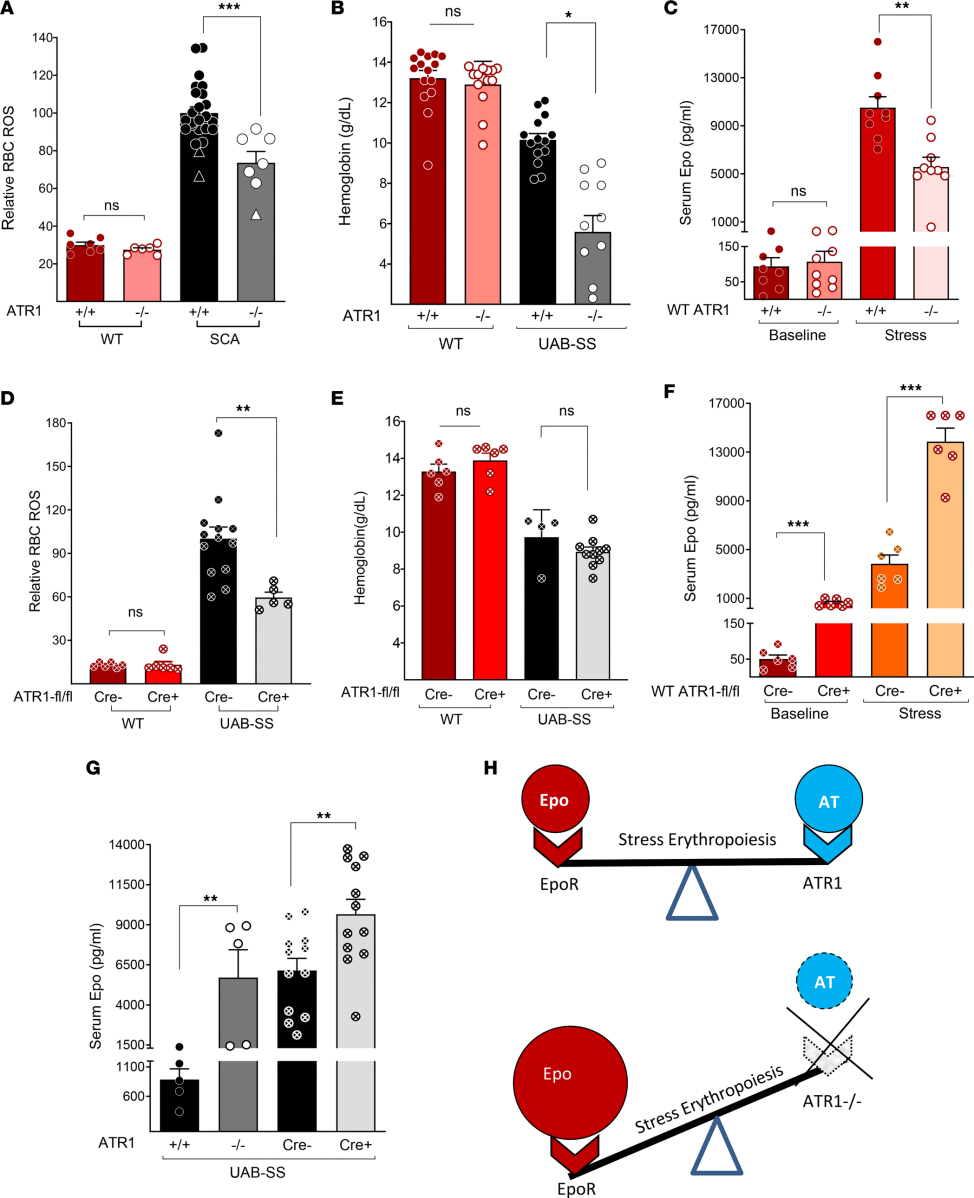
Deficient AT–ATR1 signaling lowers RBC ROS, but both AT→ATR1 signaling and Epo are necessary for stress erythropoiesis. (**A**) In unstressed WT and SCA mice with (ATR1^-/-^) and without (ATR1^+/+^) germline deletion of ATR1, the (**A**) relative RBC ROS levels (*n* = 6–7, WT mice/group. SCA ATR1^+/+^, *n* = 22 UAB-SS mice and 2 SS mice. SCA ATR1^–/–^, *n* = 6 UAB-SS mice and 1 SS mouse) and (**B**) hemoglobin levels (*n* = 14–15, WT mice/group, *n* = 11–14, UAB-SS mice/group) are shown. Circles represent UAB-SS mice and triangles represent Berkeley model) [SS] mice. (**C**) Serum erythropoietin (Epo) levels in WT ATR1*^–/–^* and WT ATR1*^+/+^* mice at baseline (unstressed) or after phlebotomy-induced erythropoietic stress (*n* = 9 mice/group). (**D** and **E**) Relative RBC ROS and hemoglobin levels in WT mice and UAB-SS mice with (ATR1^fl/fl^ Cre^+^; labeled as Cre^+^) or without (ATR1^fl/fl^ Cre^–^; labeled as Cre^–^) erythroid-specific deficiency of ATR1. WT mice *n* = 6 mice/group, UAB-SS mice *n* = 5–13 mice/group. (**F**) Serum Epo levels in WT Cre^+^ and WT Cre^–^ mice at steady state (baseline) or after phlebotomy-induced erythropoietic stress (stress; *n* = 6 mice/group). (**G**) Serum Epo levels in UAB-SS mice ATR1*^–/–^* compared with UAB-SS ATR1*^+/+^* mice (*n* = 5 mice/group) and UAB-SS mice with erythroid-specific deficiency of ATR1 (UAB-SS Cre^+^) and corresponding controls (UAB-SS Cre^–^). *n* = 12 mice/group. (**H**) Pictorial diagram showing that both AT signaling via ATR1 and Epo-EpoR signaling in erythroid cells are necessary during erythropoietic stress, and the absence of AT signaling results in a compensatory increase in Epo production. (**A** and **D**) Relative RBC ROS represents cumulative data on the MFI of CM-H_2_-DCFDA labeling in RBC with the relative change in SCA mean RBC ROS compared with the WT mean RBC ROS. Statistical analysis was done using Mann-Whitney *U* test comparing the mice with and without ATR1 deficiency. *P < 0.05, **P < 0.01, ***P < 0.001.

We measured RBC ROS and hemoglobin levels in WT mice with germline *Agtr1a* (termed ATR1 hereafter) deficiency (WT ATR1*^–/–^*) and found them similar to those in WT ATR1*^+/+^* control mice. (Figure 2, A and B). However, when we generated SCA mice (Berkeley model mice [SS] and knock-in SCA mice [UAB-SS]) with germline genetic deficiency of ATR1 (SS ATR1^–/–^ mice and UAB-SS ATR1^–/–^ mice), both SS and UAB-SS mice with ATR1 deficiency had a significant reduction in RBC ROS levels (Figure 2A) — similar to results seen with pharmacological inhibition of ATR1 signaling — suggesting that AT signaling generates ROS in sickle, but not in normal RBC. Surprisingly, germline ATR1 deficiency in SS ATR1^–/–^ mice also led to early mortality, and only an occasional SS ATR1^–/–^ mouse survived beyond weaning, with hemoglobin levels of 1–2 g/dL. The UAB-SS mice had higher baseline hemoglobin levels than the SS mice. Therefore, the anemia associated with ATR1 deficiency in them, although profound, did not cause the near-total mortality observed in SS mice, allowing measurement of hemoglobin (Figure 2B) and ROS levels in a few UAB-SS ATR1^–/–^ mice (Figure 2A). The severe anemia seen in the ATR1-deficient SCA mice was puzzling since WT mice with germline ATR1 deficiency (WT ATR1^–/–^ mice) had the same hemoglobin level as their WT ATR1*^+/+^* counterparts (Figure 2B) and had no reduction in viability or survival as compared with normal mice (WT ATR1*^+/+^* mice). Given this difference in the anemia phenotype of unstressed WT ATR1^–/–^ and UAB-SS ATR1*^–/–^* mice when compared with their respective controls, we induced stress erythropoiesis in WT ATR1*^–/–^* and WT ATR1*^+/+^* mice acutely with phenylhydrazine (which causes acute hemolysis) or subacutely (by daily phlebotomies for 3 days) and thereafter determined their hemoglobin/hematocrit levels. While at baseline/steady-state, WT ATR1*^–/–^* and WT ATR1*^+/+^* mice had similar hemoglobin levels (Figure 2B), induction of stress erythropoiesis with either phenylhydrazine or phlebotomy resulted in more exaggerated anemia in WT ATR1*^–/–^* mice compared with WT ATR1*^+/+^* mice at the peak of erythropoietic stress (Supplemental Figure 2, A and B). The recovery from the anemia, however, was similar in both groups. We also observed that while ROS levels in WT ATR1*^–/–^* and ATR1*^+/+^* RBC were similar at steady-state (Figure 2A), the RBC ROS levels were significantly lower in WT ATR1*^–/–^* mice than in control (WT ATR1*^+/+^*) mice with erythropoietic stress (Supplemental Figure 2C). These data suggest that AT→ATR1 signaling plays a critical role in maintaining hemoglobin levels during stress erythropoiesis but not in steady-state erythropoiesis. AT→ATR1 signaling generated increased ROS in sickle and stressed RBCs, a phenomenon not seen in steady-state erythropoiesis.

### AT and Epo are both necessary for stress erythropoiesis

Increased RAS signaling has been previously shown to enhance erythropoiesis by predominantly increasing Epo secretion (24). The lack of an RBC phenotype in WT ATR1*^–/–^* mice at steady-state suggested that AT signaling was largely expendable during steady-state erythropoiesis (Figure 2B). Indeed, at steady-state, Epo levels in WT ATR1*^–/–^* and WT ATR1*^+/+^* mice were comparable (averaging 101 versus 107 pg/mL) (Figure 2C).

However, in a state of stress erythropoiesis, where higher levels of Epo are necessary, intact AT→ATR1 signaling was critical, and its absence resulted in a blunted Epo response (Figure 2C). Therefore, severe anemia was seen in chronically stressed SCA ATR1*^–/–^* mice (Figure 2B) and acutely stressed WT ATR1*^–/–^* mice (Supplemental Figure 2, A and B), due to the germline lack of ATR1 signaling. Epo levels were ~100-fold higher with experimentally induced stress erythropoiesis in WT ATR1*^+/+^* mice than at steady state (Figure 2C). In contrast, WT ATR1*^–/–^* mice subjected to the same erythropoietic stress mounted only half the Epo response (~50-fold) (Figure 2C). These data show that AT’s “Epo secretagogue function” becomes relevant and rate limiting only during stress erythropoiesis, where deficiency of AT→ATR1 signaling leads to insufficient Epo production and anemia.

Since ATR1 is known to be expressed on erythroid cells (26), we wanted to assess the direct role of AT→ATR1 signaling in stress erythroid cells without the confounding effect of anemia resulting from the reduced renal “Epo secretagogue” function of AT. Therefore, we crossed WT mice with a *loxP* site inserted in the *Agtr1a* gene (known as AT1a flox strain, hereafter termed ATR1^fl/fl^), with WT mice containing the *Cre* recombinase gene that has been knocked into one allele of the Epo receptor (*Epor*) gene (known as ErGFPcre mice) (30), to generate WT mice with erythroid-specific deficiency of ATR1 (termed WT ATR1^fl/fl^ Cre^+^ mice), and the corresponding WT ATR1^fl/fl^ Cre^–^ control mice. We also generated UAB-SS mice similarly, with erythroid-specific deficiency of ATR1 (referred to as UAB-SS ATR1^fl/fl^ Cre^+^) and the corresponding controls with intact ATR1 on erythroid cells (UAB-SS ATR1^fl/fl^ Cre^–^ mice).

WT ATR1^fl/fl^ Cre^+^ mice showed no difference in RBC ROS or hemoglobin levels compared with their respective controls at steady-state (Figure 2, D and E), data similar to that of unstressed germline WT ATR1*^–/–^* mice and their respective control WT ATR1*^+/+^* mice (Figure 2, A and B). However, WT ATR1^fl/fl^ Cre^+^ mice subjected to stress erythropoiesis with phlebotomy had lower RBC ROS levels than WT ATR1^fl/fl^ Cre^–^ mice (Supplemental Figure 2D), although their hemoglobin/hematocrit levels were similar (Supplemental Figure 2, E and F). Similarly, UAB-SS ATR1^fl/fl^ Cre^+^ mice, which are in a perpetual state of stress erythropoiesis, when compared with their UAB-SS ATR1^fl/fl^ Cre^–^ counterparts, had lower RBC ROS levels (Figure 2D) but similar hemoglobin levels (Figure 2E). This contrasted with the profound anemia observed in SCA mice with germline deficiency of ATR1 (SS ATR1*^–/–^* and UAB-SS ATR1*^–/–^*), in which the Epo secretagogue function of AT was affected (Figure 2, B versus E).

Epo levels in WT mice with erythroid-specific deficiency of ATR1 (WT ATR1^fl/fl^ Cre^+^ mice) were nearly 12-fold higher even at baseline/steady-state, compared with WT ATR1^fl/fl^ Cre^–^ mice (Figure 2F). The increased Epo signaling at steady state fully compensated for the AT signaling deficiency in erythroid cells, maintaining normal hemoglobin levels (Figure 2E). Furthermore, WT ATR1^fl/fl^ Cre^+^ mice subjected to stress erythropoiesis exhibited remarkably higher Epo levels than WT ATR1^fl/fl^ Cre^–^ mice (13,861 pg/mL in WT ATR1^fl/fl^ Cre^+^ mice versus 3,838 pg/mL in WT ATR1^fl/fl^ Cre^–^ mice), thereby preventing anemia (Figure 2F). This was also reflected in a higher reticulocyte response around the peak erythropoietic stress in WT ATR1^fl/fl^ Cre^+^ mice compared with WT ATR1^fl/fl^ Cre^–^ mice subjected to the same phlebotomy-induced stress (Supplemental Figure 2G). Hence, during stress erythropoiesis, the lack of AT signaling in erythroid cells is compensated for by enhanced renal Epo secretion (which can readily occur when ATR1 signaling is intact in all other cells except erythroid cells), preventing the development of anemia. However, this compensatory increase in Epo production during erythroid stress cannot occur when AT signaling is globally deficient in germline ATR1-deficient mice (Figure 2, C versus F, and Supplemental Figure 2, A and B versus E and F), resulting in anemia.

The same phenomenon was observed in UAB-SS mice, which are in a state of chronic stress erythropoiesis. Global deficiency of AT signaling in UAB-SS mice resulted in profound anemia, as these mice were unable to increase Epo secretion to sufficient levels to compensate for the lack of Epo secretagogue function of AT (Figure 2, B and G). On the other hand, when AT→ATR1 signaling was only deficient in erythroid cells, UAB-SS ATR1^fl/fl^ Cre^+^ mice were able to increase Epo secretion to remarkably high levels and, therefore, did not show a decline in hemoglobin compared with UAB-SS ATR1^fl/fl^ Cre^–^ mice (Figure 2, E and G).

It was notable, however, that increased AT→ATR1 signaling in erythrocytes produced by stress, but not steady-state erythropoiesis, led to increased RBC ROS production, both in WT mice (Supplemental Figure 2, C and D) and UAB-SS mice (Figure 2, A and D). The interruption of AT→ATR1 signaling, either globally or only in erythroid cells, reduced RBC ROS levels. However, in UAB-SS ATR1*^–/–^* mice, the ROS levels did not return to the level seen in WT mice, likely because sickle hemoglobin generates ROS nonenzymatically via the Fenton reaction (11).

Our data suggest that both AT→ATR1 signaling and Epo are necessary to increase erythroid cell production during erythropoietic stress (Figure 2H). However, unlike Epo-EpoR, the AT→ATR1 pathway in stress erythrocytes led to significant ROS production, and the ROS-RAS signaling loop perpetuated itself in SCA.

We had previously shown that excessive AT production mediated sickle cell nephropathy via ATR1 (1). Herein, we demonstrate that SCA mice with erythroid-specific deficiency of ATR1 signaling did not develop albuminuria, a key feature of sickle nephropathy (Supplemental Figure 3), suggesting that ROS-RAS signaling driven by sickle RBC mediates nephropathy as a bystander effect.

### Mechanisms underlying increased ROS generated by AT signaling in RBCs

#### AT does not induce ROS via canonical NOX signaling in stress erythrocytes.

AT→ATR1 signaling canonically generates ROS intracellularly via NOX in various nonerythroid cells (31, 32). We subjected WT and SS RBC in vitro to diphenyleneiodonium (DPI; a NOX inhibitor) and a variety of other ROS production inhibitors, including rotenone (a selective inhibitor of complex I of the mitochondrial respiratory chain). Both inhibition of NOX (by DPI) and mitochondrial ROS (by rotenone) significantly reduced ROS in vitro (Supplemental Figure 4A). Surprisingly, in SCA mice (both SS mice and UAB-SS mice), blockade of NOX signaling with apocynin (a NOX inhibitor) in vivo did not reduce sickle RBC ROS (Supplemental Figure 4, B and C). DPI and Apocynin are nonspecific NOX inhibitors; given the discrepancy between our in vitro assay using DPI and our in vivo experiments using Apocynin, we generated UAB-SS mice genetically deficient in p22phox (hereafter referred to as gp22^–/–^). The p22phox protein is a common subunit in NOX 1–4, resulting in the inactivation of all isoforms of NOX (Supplemental Figure 4D). While WT gp22^–/–^ mice had significantly lower RBC and retic ROS levels, UAB-SS gp22^–/–^ mice showed no reduction in RBC or reticulocyte ROS (Supplemental Figure 4, E and F). These data suggest that AT→ATR1 signaling engages NOX in RBCs and generates ROS during steady-state erythropoiesis in WT gp22^–/–^ mice. However, this canonical NOX signaling was not the major contributor to RBC ROS production during stress sickle erythropoiesis in UAB-SS gp22^–/–^ mice.

#### AT→ATR1 signaling promoted the accumulation of dysfunctional mitochondria that generate high ROS in erythrocytes.

Mitochondria are a major source of intracellular ROS production, and AT plays a role in mitochondrial function (20, 33). However, the underlying molecular mechanism by which AT→ATR1 signaling stimulates mitochondrial ROS is unclear. We first determined the effect of deficient AT signaling on mitochondrial mass (through TOM20 labeling) in both stressed nucleated and enucleated erythroid cell populations obtained from the spleen (site of stress erythropoiesis in mice). The gating technique used to identify nucleated (erythroid precursors) and enucleated (reticulocytes and RBCs) erythroid subpopulations is described in the methods section and shown in Supplemental Figure 5 (34).

**Figure 3:**
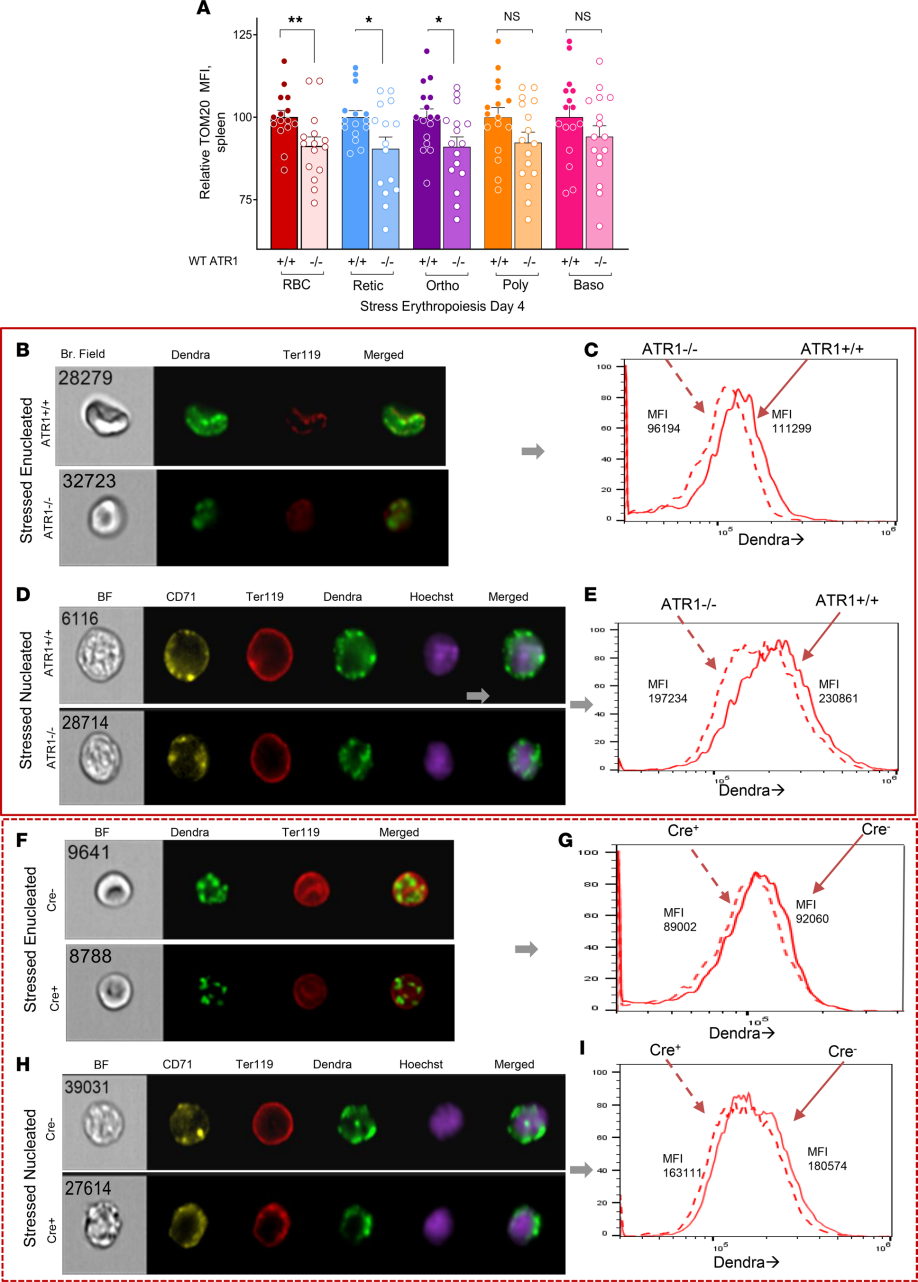
Angiotensin signaling promotes mitochondrial retention during stress erythropoiesis. (**A**) Cumulative data on the MFI of TOM20-labeled erythroid subpopulations acquired 4 days after phenylhydrazine (PHZ) administration in ATR1 germline-deficient ATR1*^–/–^* mice (light shaded bars) versus control ATR1*^+/+^* mice (dark shaded bars); red, RBC; blue, reticulocyte; purple, orthochromatic; yellow, polychromatophilic; pink, basophilic compartments (*n* = 15 mice each). Statistical analysis was performed using multiple *t* tests to compare the respective erythroid subpopulations. *P < 0.05, **P < 0.01. (**B**–**E**) Representative figures from image stream (IS) analysis done on PHZ-induced stressed Mito-Dendra2 (mitochondria show green fluorescence; labelled as Dendra) ATR1^–/–^ mice compared with that of control Mito-Dendra2 ATR1*^+/+^* mice. Images were acquired at the peak of erythropoietic stress, 4 days after PHZ administration. Enucleated erythrocyte (Hoechst^-^Ter119^+^) (**B**) and nucleated precursors (Hoechst^+^Ter119^+^) (**D**) are shown. Mito-Dendra MFI of these cells is plotted in **C** and **E**, respectively, where the MFI of ATR1^–/–^ (dashed lined) and ATR1^+/+^ (solid lined) cells are listed within the histograms. (**F**–**I**) Representative figures from IS analysis done in PHZ-induced stressed Mito-Dendra2 ATR1^–/–^ mice with (Cre^+^) or without (Cre^–^) erythroid-specific deficiency of ATR1. Enucleated (**F**) and nucleated erythroid precursors (**H**) are shown. The Mito-Dendra MFI is plotted in **G** and **I**. The MFI values of Cre^+^ (dashed-lined histograms) and Cre^–^ (solid-lined histograms) cells are listed within the histograms. We acquired a minimum of 10,000 cells/mouse on IS from each animal, with a total of 2–3 mice/group. The number shown on the top left inside the bright-field images in **B**, **D**, **F**, and **H** is the unique identification number of that particular cell.

At the peak of acute hemolysis-induced stress erythropoiesis with phenylhydrazine (on Day 4 after administration) (Figure 3A) or phlebotomy (after 3 days of daily phlebotomy) (Supplemental Figure 6A), WT ATR1*^–/–^* mice had significantly lower mitochondrial mass in the late erythroid precursors and enucleated erythroid cells (reticulocytes and RBC), while early erythroid precursors also tended to have lower mitochondrial mass than their corresponding erythroid populations in ATR1*^+/+^* mice. These findings of reduced mitochondrial mass in ATR1*^–/–^* stress erythroid cells were confirmed by Image-stream analysis of TOM20-stained nucleated and enucleated erythroid populations (Supplemental Figure 6, B–E).

We also bred ATR1*^–/–^* mice with Mito-Dendra2 mice (which have eGFP-labeled fluorescent mitochondria) to generate Dendra ATR1*^–/–^* mice and compared them to Dendra ATR1*^+/+^* (control) mice subjected to phenylhydrazine stress. Dendra ATR1*^–/–^* had a lower mitochondrial mass in their stressed enucleated (Figure 3, B and C) and nucleated (Figure 3, D and E) erythroid populations compared with their respective controls (Dendra ATR1*^+/+^* mice). Furthermore, Mito-Dendra2 mice with erythroid-specific deficiency of ATR1 (termed Dendra Cre^+^) also had fewer mitochondria in the stressed enucleated (Figure 3, F and G) and nucleated erythroid cell populations (Figure 3, H and I). This phenomenon was seen only during erythropoietic stress. Before stress induction, there was no difference in mitochondrial content (when labeled with TOM20 or with eGFP) between ATR1*^+/+^* and ATR1*^–/–^* erythroid cells as seen by Image-stream analysis (Supplemental Figure 7, A–D). These data confirm that ATR1 deficiency (either germline or erythroid-specific) reduced mitochondrial content in erythroid (nucleated and enucleated) populations and that increased AT→ATR1 signaling in erythroid cells during stress erythropoiesis promoted higher mitochondrial mass, likely due to the high mitotic and metabolic demand during stress erythropoiesis.

**Figure 4:**
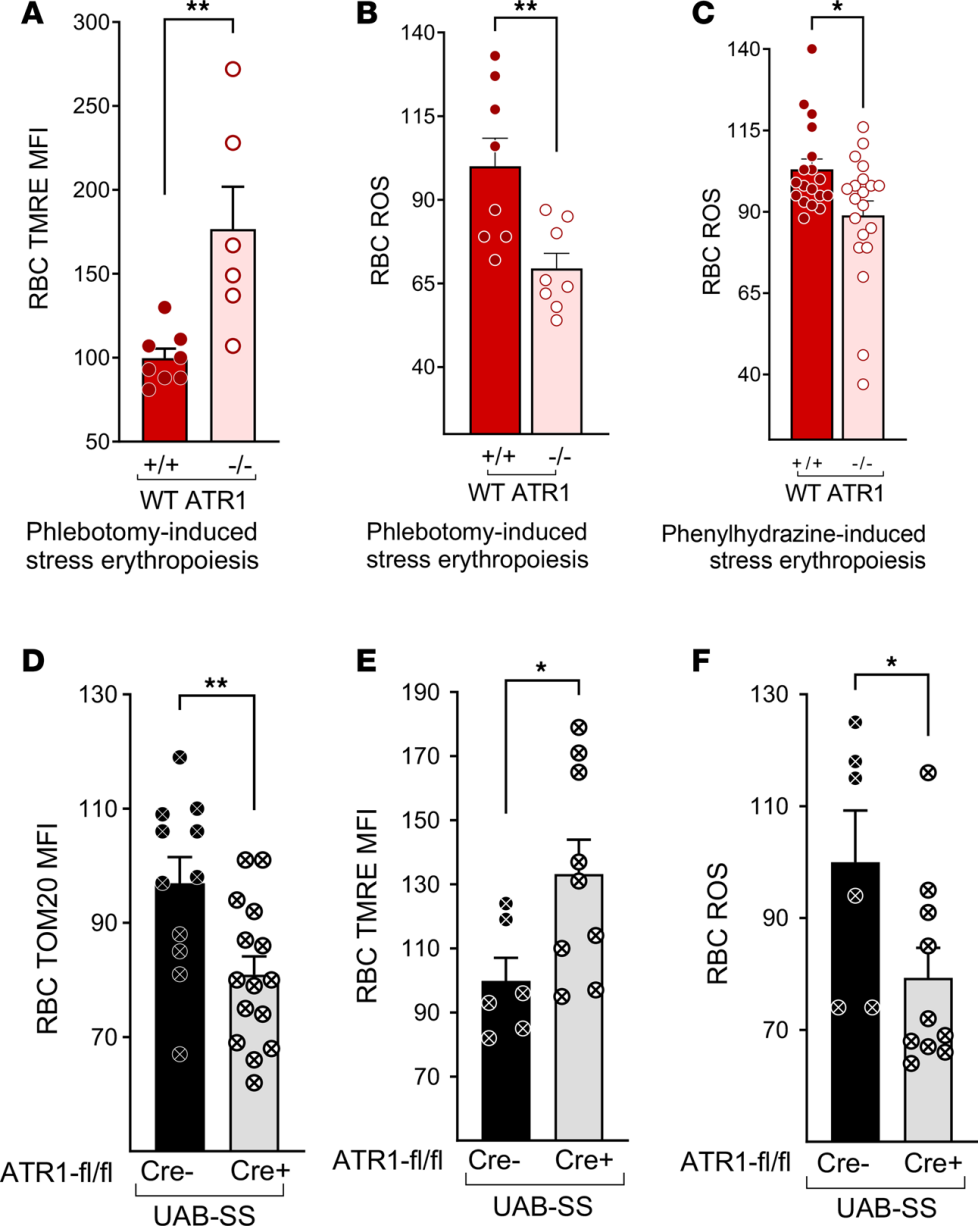
Angiotensin signaling in stress erythropoiesis promotes the accumulation of dysfunctional mitochondria, which generate high levels of reactive oxygen species (ROS) in erythrocytes. (**A** and **B**) RBC from spleens from WT *Agtr1a* (labeled as ATR1)^+/+^ and ATR1^–/–^ mice stressed with daily phlebotomy were stained forTMRE mean fluorescence intensity (MFI) reflecting mitochondrial membrane potential (**A**), and ROS (relative CM-H_2_-DCFDA MFI) (**B**) (*n* = 5–8 mice/group). (**C**) RBC ROS (relative CM-H_2_-DCFDA MFI) in WT ATR1*^+/+^* and ATR1^–/–^ mice stressed with phenylhydrazine (*n* = 18–20 mice/group). (**D**–**F**) Bone marrow RBC from UAB-SS mice with (UAB-SS ATR1^fl/fl^ Cre^+^; labeled as Cre^+^) and without (UAB-SS ATR1^fl/fl^, Cre^–^; labeled as Cre^–^) erythroid-specific deficiency of ATR1 stained for TOM20 (representing mitochondrial mass) (**D**), TMRE (representing mitochondrial membrane potential) MFI (**E**), and ROS (relative CM-H_2_-DCFDA MFI) (**F**) (*n* = 6–15 mice/group). Statistical analysis was done using Mann-Whitney *U* test. *P < 0.05, **P < 0.01.

Next, we determined the mitochondrial membrane potential using tetramethylrhodamine, ethyl ester. We postulated that the excess mitochondria, especially those retained in stressed RBCs resulting from AT→ATR1 signaling, were dysfunctional, with a depolarized or lower mitochondrial membrane potential, contributing to the high ROS levels. Indeed, ATR1*^+/+^* RBCs from phlebotomy-induced stress erythropoiesis exhibited a lower mitochondrial membrane potential and higher ROS compared with ATR1*^–/–^* RBCs (Figure 4, A and B). Similarly, lower ROS levels were also seen in ATR1*^–/–^* RBCs following acute hemolytic stress induced by phenylhydrazine (Figure 4C). The lower ROS levels were also noted in the nucleated erythroid precursors (orthochromatic, polychromatophilic, basophilic) of stressed (phenylhydrazine and phlebotomy) ATR1*^–/–^* mice (Supplemental Figure 8, A and B). Phenylhydrazine administration caused technical interference with TMRE staining; therefore, mitochondrial membrane potential in phenylhydrazine-stressed RBCs could not be evaluated.

SCA mice exhibit chronic stress erythropoiesis to compensate for ongoing hemolysis, although this is still insufficient to prevent anemia. SCA mice with germline ATR1 deficiency could not be assessed due to high mortality from severe anemia. However, sickle RBC in mice with erythroid-specific deficiency of ATR1 (UAB-SS ATR1^fl/fl^ Cre^+^ mice) had lower mitochondrial mass, higher mitochondrial membrane potential, and lower ROS (Figure 4, D–F) than their corresponding UAB-SS ATR1^fl/fl^ Cre^–^ controls, suggesting the presence of fewer dysfunctional mitochondria in SCA mice deficient in AT signaling in erythroid cells.

Taken together, our results demonstrate that AT→ATR1 signaling plays a critical role in enhancing erythropoiesis during stress states. While it boosted mitochondrial content accompanying this erythroid-cell expansion, it also led to the retention of dysfunctional mitochondria, resulting in higher ROS levels in erythroid cells.

The experimental schematic clarifying the rationale and experimental strategy for each set of data in the results section is shown in Supplemental Figure 9.

## Discussion

In this study, we first explored the mechanisms underlying hyperangiotensinemia in SCA and found that elevated erythroid-cell ROS increased AT production. We and others have previously demonstrated that high ROS levels in sickle RBCs increase RBC fragility, hemolysis, and ROS release into the plasma (11, 12, 35), thereby promoting SCA-associated vasculopathy (4, 35). High oxidative stress/ROS in preeclampsia has been shown to increase oxidized ATGN, resulting in higher AT production and hypertension (3). We therefore postulated that the high RBC ROS in SCA may promote elevated AT levels via this mechanism. In this study, we show that in SCA mice, oxidized ATGN, plasma AT, and RBC ROS levels were high (Figure 1). Blockade of AT→ATR1 signaling in SCA mice, either pharmacologically (captopril or losartan) or via a germline ATR1 KO, resulted in a significant reduction in RBC ROS and reduced RAS pathway activation. These findings indicate that increased AT→ATR1 signaling in sickle erythroid cells generates high levels of ROS, thus creating a positive feedback loop of ↑ROS→ ↑oxidized ATGN→ ↑AT-ATR1→ ↑ROS, which causes and perpetuates the hyperangiotensinemia. This feedback loop appears to be driven primarily by SCA hematopoiesis, as WT mice transplanted with SCA bone marrow developed high ROS and AT levels, which decreased with RAS inhibition. Furthermore, we previously showed that AT→ATR1 signaling promotes sickle nephropathy and that global ATR1 blockade (pharmacological or germline deficiency) decreased albuminuria (1). Herein, we show that sickle erythroid cells appeared to be driving organ damage, as sickle mice with erythroid-specific ATR1 deficiency had amelioration of albuminuria, a prominent feature of sickle nephropathy.

In previously reported in vitro studies, AT→ATR1 signaling has been shown to stimulate the proliferation of human erythroid progenitors (26), while the addition of ACE-I and ATR1 blockers reduced erythroid colony formation in healthy and chronic hemodialysis patients (36). However, in vivo studies contradicted these results. When ATR1-KO mice were generated, they had no RBC phenotype (24). Herein, we also showed that at steady-state erythropoiesis, WT mice with germline or erythroid-specific deficiency of AT signaling were not anemic. These murine findings are further supported by multiple human studies reporting a clinically negligible (~0.5 g/dL) decrease in hemoglobin with RAS inhibitor use (37). Therefore, data from murine and human studies suggest that AT signaling plays a relatively minor role in steady-state erythropoiesis; a testament to this is the widespread clinical use of AT and ATR1 blockers. Furthermore, the effect of AT on erythropoiesis appears to differ between healthy and diseased states, as patients who are susceptible to anemia with RAS inhibition are those with renal or heart failure, who depend more on angiotensin for erythropoiesis maintenance (38–40).

The majority of studies on RAS inhibitors in patients with SCA have focused on evaluating their effects on SCA-related renal injury, primarily albuminuria and glomerular filtration rate. While a decline in hemoglobin concentration was noted in some of these studies, it ranged from 0.3 to 0.6 g/dL and did not lead to discontinuation or dose reduction of the drug (41–44). In a recently presented abstract, Saraf and colleagues evaluated the effect of RAS inhibitors on hemoglobin concentration in a cross-sectional analysis of the WALK-PHaSST cohort (45), a longitudinal analysis of the University of Chicago (UC; Chicago, Illinois, USA) registry, and a Multicenter Phase 2 Losartan study (44). RAS inhibitor use was associated with a statistically significant reduction in hemoglobin concentration of 0.4 g/dL (46). Although this decrease is small, we need to be cautious when administering these medications to patients with SCA who are already severely anemic and monitor them closely, as they may not tolerate any further drop in hemoglobin and may need periodic transfusions. The anemia seen in the SCA mouse model is higher with RAS inhibition because erythrocyte turnover is much higher in SCA mice than in humans with SCA.

In our study, we further explored a unique role of AT→ATR1 signaling in stress erythropoietic states. SCA mice or stressed WT mice with germline ATR1 deficiency developed profound anemia. We observed that AT→ATR1 signaling acted in concert with Epo→EpoR signaling and was necessary to maintain stress erythropoiesis. At steady-state, WT ATR1^fl/fl^ Cre^+^ mice had higher Epo levels than their respective WT ATR1^fl/fl^ Cre^–^ controls. It is to be noted that WT ATR1^fl/fl^ Cre^+^ mice were generated by knocking in GFP-Cre into one of the alleles of *Epor*. High Epo levels in these mice could be compensatory to the haploinsufficiency of *Epor*. However, it has been shown that 40% EpoR expression (*Epor-*null mice with transgene-derived *Epor* expression) results in a 1.5- to 2-fold increase in Epo levels; at best, both at baseline and under stress-induced erythropoiesis (47). We observed a 12-fold higher Epo expression in WT ATR1^fl/fl^ Cre^+^ mice compared with WT ATR1^fl/fl^ Cre^–^ mice at baseline, which suggests that AT signaling probably plays a role in steady-state erythropoiesis to adequately compensate for erythropoiesis in WT ATR1^fl/fl^ Cre^+^ mice by increasing Epo levels. In stress erythropoietic states (acute anemia or chronic anemia, as seen in SCA), AT→ATR1 appeared to play a substantially greater role, as Epo levels in stressed WTATR1^fl/fl^ Cre^+^ mice were exponentially higher compared with WTATR1^fl/fl^ Cre^–^ mice to compensate for the deficient AT signaling. Furthermore, in contrast to steady-state erythropoiesis, stress erythropoiesis in WT or SCA mice with germline deficiency of ATR1 resulted in profound anemia, leading to mortality in SS ATR1*^–/–^* mice, which were unable to increase Epo production to a level required to sustain adequate erythropoiesis. Stressed WT ATR1^fl/fl^ Cre^+^ or UAB-SS ATR1^fl/fl^ Cre^+^ mice were able to increase Epo levels to compensate for the interruption in AT→ATR1 signaling and maintain hemoglobin levels. It is worth noting that recent studies have shown that EpoR-Cre is not strictly erythroid specific, as hematopoietic stem cells, megakaryocytes, and macrophages can also express EpoR (48). With the demonstration of EpoR expression on nonerythroid cells as well, future studies using a more erythroid-specific mouse model can investigate whether AT signaling via nonerythroid cells affects erythropoiesis.

Retention of mitochondria in RBCs and reticulocytes of phlebotomized mice and SCA mice has been previously reported (12, 13). We found that 1 mechanism underlying retained mitochondria in stressed enucleated (reticulocytes and RBCs) and nucleated erythroid cells is from AT→ATR1 signaling, wherein ATR1*^–/–^* erythroid cells (both by flow cytometry for mitochondria and ImageStream analysis of GFP-labeled mitochondria) had decreased mitochondrial mass compared with stressed ATR1*^+/+^* erythroid cells. The lower mitochondrial mass in the stressed ATR1*^–/–^* mice also corresponded to the decreased overall erythroid cell mass, as evidenced by the lower hematocrit. Taken together, our data suggest that AT→ATR1 signaling likely played an essential role in stress erythropoiesis by maintaining erythroid cell and mitochondrial mass.

We finally show that excessive ROS production was a bystander effect of AT→ATR1 signaling in stress erythropoiesis. AT→ATR1 signaling is known to generate ROS intracellularly via NOX in various nonerythroid cell types (31, 32). We had previously shown the presence of NOX signaling in erythroid cells (11). However, herein we found that pharmacological inhibition or a genetic deficiency of NOX did not decrease the RBC ROS levels in SCA mice. Hence, NOX activation was not the primary source of ROS generation by AT signaling in stress erythroid cells. Mitochondria are the seat of cellular respiration and are necessary to sustain the increased erythroid cell production required in stress erythropoiesis. Mitochondria-retaining RBCs and reticulocytes in SCA mice and WT stress-induced anemic mice exhibit higher ROS levels (12, 13). Our study shows that AT signaling increased erythroid mitochondrial mass and promoted mitochondrial dysfunction in stressed WT and sickle erythroid cells, thereby increasing erythroid ROS.

To the best of our knowledge, the critical role of AT signaling in erythropoiesis — and its indispensable role in stress erythropoiesis, highlighted in this study — has not been previously reported. AT→ATR1 signaling in sickle erythrocytes appears to be a double-edged sword. On one hand, it plays a critical role in sustaining enhanced erythropoiesis and increased mitochondrial mass, which are essential in erythropoietic stress states. On the other hand, it increased dysfunctional mitochondria and ROS levels in the circulating sickle RBC, further accentuating RAS signaling. This positive feedback loop, essential for sustaining sickle erythropoiesis, also has detrimental bystander effects of increased RAS signaling, which manifests as nephropathy. Blocking AT→ATR1 signaling alone may be sufficient to reduce ROS, RAS, and sickle cell nephropathy.

## Methods

### Sex as a biological variable.

For our study, sex was not used as a biological variable.

### Experimental mice.

Berkeley model (termed SS), C57BL/6N*-Agtr1a^tm1Uky^/J* mice (termed ATR1^fl/fl^), and p22phox-deficient mice, A.B6 *Tyr^+^- Cyba^nmf333^*/J (termed gp22^–/–^) were obtained from Jackson Laboratory. Townes model (termed UAB-SS), ErGFPcre mice, and Mito-Dendra2 mice (in which Cox8, a mitochondrial outer membrane protein, was targeted with eGFP dendra protein, making mitochondria fluorescently labeled) were provided by Timothy Townes (University of Alabama, Birmingham, Alabama, USA), Ursula Klingmüller (German Cancer Research Center, Heidelberg,Germany) (30), and Marie-Dominique Filippi (Cincinnati Children’s Hospital Medical Center, Cincinnati), respectively. The ErGFPcre mice are a knock-in mouse model that expresses a GFP-Cre fusion protein controlled by the endogenous *Epor* promoter and, therefore, are haploinsufficient for *Epor*. The GFP expression in these mice is negligible. Genetic crosses (including UAB-SS ATR1*^–/–^*, UAB-SS ATR1^fl/fl^ Cre^+^, UAB-SS gp22^–/–^, Dendra ATR1*^–/–^*, Dendra Cre^+^) were made by crossbreeding, with details provided in the Supplemental Methods. Sickle chimeric mice were obtained via transplant as previously described (1, 2, 49).

### Drug treatments.

Mice were treated for 3–6 months with the following drugs in drinking water: Captopril (West-Ward Pharmaceutical) 0.15 mg/mL; Losartan (Teva Pharmaceuticals) 0.3–0.6 mg/mL, Apocynin (4′-Hydroxy-3′-methoxyacetophenone, SIGMA, Catalog # A10809) 1 mg/mL. SS mice were placed on captopril, losartan, or apocynin at 8–12 weeks of age for 3–6 months. Drug treatment was initiated in the chimeric mice 3 months after the bone marrow transplant, when the mice had achieved full donor (sickle) chimerism, and continued for 6–9 months.

Stress erythropoiesis induction was performed either to cause acute hemolysis with 100 mg/kg phenylhydrazine in phosphate-buffered saline injected i.p., or subacutely, by daily phlebotomy (500 μL from the tail vein) for 3 days.

### Flow-cytometry and imaging flow cytometry (IFC) analysis.

Mouse blood, spleen, and bone marrow were analyzed for erythroid cells for ROS levels, mitochondrial mass, and membrane potential (50). The distribution of mitochondria was studied using IFC with Amnis ImageStreamX analysis. At least 10,000 events per sample were collected and analyzed with the associated Image Data Exploration and Analysis Software (IDEAS; Amnis) at 40×/numerical aperture 0.75 and 60×/numerical aperture 0.9 objective lenses.

### ATGN and AT analysis.

The redox status of ATGN was determined after polyethylene-glycol (PEG5000 maleimide) adduct formation, followed by Western blot analysis with an anti-AGT antibody. Urine AT levels were measured by using the Angiotensin II EIA kit (Cayman Chemicals).

Methodological details are provided in the Supplemental Methods. The experimental schematic outlining the experimental strategy and rationale for all the experiments conducted is shown in Supplemental Figure 9.

### Statistics.

was performed using GraphPad Prism version 6. The results are presented as mean ± SEM. Data between experimental groups of mice were analyzed using nonparametric 1- or 2-tailed *t* tests, Mann Whitney U test, or 1-way or 2-way ANOVA, as indicated in the figure legends; *P* values are shown for each analysis. *P* values less than 0.05 were considered statistically significant.

### Study approval.

The experimental mice were maintained in the Cincinnati Children’s Research Foundation vivarium, following IACUC-approved protocols (IACUC protocol nos. 2E07051, 2D07050, 2015-0076). The study on human samples was conducted in compliance with the Declaration of Helsinki and with policies approved by the IRB of Cincinnati Children’s Hospital Medical Center, Cincinnati, Ohio (protocol no. 2008-0304).

### Data availability.

Data supporting the findings of this study are available within the article, supplemental material, and Supporting Data Values. Any remaining data can be made available from the corresponding author upon reasonable request.

## Author contributions

PR, SR, DGK, PA, MSEMM, AS, and SRP performed the experiments and analyzed, plotted, and interpreted the data. TAK designed the NOX experiments, generated the *Cyba^nmf333^* sickle mice in her laboratory, and provided oversight in the analysis and interpretation of the NOX data. PM conceived the project, designed the experiments, and interpreted the data. PR and PM wrote the manuscript. All authors reviewed and edited the manuscript.

## Conflict of interest

The authors have declared that no conflict of interest exists.

## Funding support

This work is the result of NIH funding, in whole or in part, and is subject to the NIH Public Access Policy. Through acceptance of this federal funding, the NIH has been given a right to make the work publicly available in PubMed Central.

- Excellence in Hemoglobinopathy Research Award (EHRA).
- U01HL117709 (PM).
- NIH-NHLBI R34 HL108752 (PM).
- Academic Research Council Grant, CCHMC (PM).
- PR was the Translational Research Scholar on the EHRA U01HL117709.

## Supplementary Material

Supplemental data

Unedited blot and gel images

Supporting data values

## Version 1 03/20/2026

In-Press Preview

## Version 2 05/08/2026

Electronic publication
