# Supplementary material for: Angiotensin signaling is essential for stress erythropoiesis but causes retention of dysfunctional mitochondria in RBCs
Source: JCI Insight. 2026 Mar 12;11(9):e200722. doi: 10.1172/jci.insight.200722 (PMC13232005; doi:10.1172/jci.insight.200722)
Supplement: Supplemental data [file jciinsight-11-200722-s058.pdf]

Supplemental Methods

Experimental mice

All mice used in experiments were 6-16 weeks of age. Berkeley model mice (Tg[Hu-miniLCR$\alpha 1^{G}\gamma^{A}\gamma\delta\beta^{S}$] *Hba*$^{0/0}$ *Hbb*$^{0/0}$) (1) that exclusively express human sickle hemoglobin are termed as SS mice and were obtained from Jackson Laboratory (Bar Harbor, Maine). Wild type mice (C57Bl/6) referred as WT, germline ATR1 deficient mice (*Agtr1a*$^{-/-}$) termed as ATR1$^{-/-}$, and p22phox-deficient mice (A.B6 *Tyr*$^{+}$-*Cyba*^{nmf333}/J) termed as gp22$^{-/-}$ (2) were also obtained from Jackson Laboratory. Knock-in sickle mice (B6;129-*Hba*$^{tm1(HBA)Tow}$*Hbb*$^{tm2(HBG1,HBB^{*})Tow}$/*Hbb*$^{tm3(HBG1,HBB)Tow}$/J) in which the human α and β^{S} globin genes have been knocked in to replace the mouse α and β (β^{major} and β^{minor}) globin genes, respectively, were kindly provided by Dr. Timothy Townes (University of Alabama [UAB], Birmingham) and are termed as UAB-SS mice. As UAB-SS females are infertile, female mice that have one normal human β-globin allele and one β^{S} allele (UAB-AS) were bred with UAB-SS male mice to obtain UAB-SS mice for experiments and with UAB-AS males to obtain the UAB-AA normal counterparts.

Erythroid-specific WT ATR1-deficient mice (*Agtr1a*$^{fl/fl}$ *Epor-Cre*), termed as WT ATR1 fl/fl Cre+, were generated by crossing the Jackson Laboratory strain C57BL/6N-*Agtr1a*^{tm1Uky}/J mice (known as AT1 flox strain, termed hereafter as ATR1 fl/fl) with C57Bl/6 mice containing the *Cre* recombinase gene that has been knocked into one allele of the erythropoietin receptor (*Epor*) gene known as ErGFPcre mice (kindly provided by Dr. Ursula Klingmüller; German Cancer Research Center). (3) Hence, it should be noted that WT ATR1 fl/fl Cre+ mice express EpoR from only one allele and Cre recombinase from the other allele.

SCA mice with germline or erythroid-specific ATR1 deficiency: Berkeley model (SS) mice were bred with WT ATR1$^{-/-}$ mice and backcrossed to SS mice several times until in males, mouse globin genes acquired by breeding with WT ATR1$^{-/-}$ mice were eliminated and the human α and β^{S} globin transgene retained, along with the *Agtr1a* knockout allele to derive SS ATR1$^{-/-}$ mice. For female breeders, one copy of the mouse β-globin and the human α and β^{S} globin transgene was retained along with *Agtr1a* knockout alleles, since female (but not male) SCA mice are infertile. A high mortality was seen in SS ATR1$^{-/-}$ mice by 4-5 weeks of age from severe anemia. Hence, UAB-SS mice, which had higher baseline hemoglobin, were interbred with ATR1$^{-/-}$ mice to derive UAB-SS ATR1$^{-/-}$ mice with human α and β^{S} globin alleles and *Agtr1a* knockout alleles.

Using a similar strategy to derive SCA mice with germline deficiency of ATR1, erythroid-specific ATR1-deficient sickle mice (UAB-SS *Agtr1a*$^{fl/fl}$ *Epor-Cre)*, termed as UAB-SS ATR1 fl/fl Cre+, were generated as follows: UAB-SS mice were bred with ATR1 fl/fl mice and then with ErGFPcre mice, and then back-crossed until mouse globin genes were eliminated.

WT mice deficient in p22phox (A.B6 *Tyr*$^{+}$-*Cyba*^{nmf333}/J), termed WT gp22$^{-/-}$mice, were bred with UAB-SS mice to obtain UAB-SS gp22$^{-/-}$ mice after backcrossing to eliminate mouse globin genes.

Mito-Dendra2 mice (B6;129S-*Gt(ROSA)26Sor*$^{tm1.1(CAG-COX8A/Dendra2)}$), termed as Dendra, in which cox8, a mitochondrial outer membrane protein, was targeted with eGFP Dendra protein, making mitochondria fluorescently labeled, were kindly provided by Dr. Marie-Dominique Filippi (Cincinnati Children's Hospital Medical Center).(4) Dendra mice were bred with WT ATR1$^{-/-}$ mice to generate Dendra ATR1$^{-/-}$mice, which have global ATR1 deficiency. Dendra mice with

erythroid-specific deficiency of ATR1, termed Dendra Cre+, were derived by breeding Dendra mice first with the ATR1 fl/fl strain and then with ErGFPcre mice.

To generate the different sickle chimeric mice described in this study, we transplanted lethally irradiated (1,250 cGy) BL/6.Boy J (CD45.1) recipient mice at 8-10 weeks of age with bone marrow from either SS or C57BL/6 (CD45.2) mice in a 1:6–7 donor to recipient ratio as described previously. (5-8) Only mice showing complete donor chimerism (>99% donor, determined via HPLC for sickle hemoglobin or by FACS for CD45.1 for sickle and WT chimeras, respectively) at 3 months post-transplant were considered evaluable for experiments, as previously described. The nomenclature used for the chimeric mice was donor genotype/recipient genotype (e.g., SS/WT: SS donor bone marrow transplanted into WT recipient mice, resulting in mice fully chimeric for SS hematopoiesis). The sickle donor chimeras developed hematological parameters characteristic of SS mice. Peripheral blood from the experimental mice was collected from the tail vein or the retro-orbital sinus. Hematology parameters were analyzed using an automated analyzer (Hemavet, Drew Scientific).

Flow cytometry*:*

Mouse blood, spleen, and bone marrow were analyzed for erythroid precursor subpopulations and enucleated erythrocytes for reactive oxygen species (ROS) levels, mitochondrial membrane potential, and mitochondrial mass.(9)

Blood samples were stained with APC-conjugated anti-Ter119 (Biolegend,116212), PE-Cy7-conjugated anti-CD45 (BD Biosciences, 55774), and PE-conjugated anti-CD41 (BD Biosciences, 553267) antibodies for 30 min at RT to identify single RBC and WBC populations and exclude platelets after gating cells based on specific forward and side scatter properties. Cell suspension of spleen and bone marrow specimens were stained with APC-conjugated anti-Ter119 (Biolegend,116212), APC-Cy7-conjugated-anti-CD44 (BD Biosciences, 560568), PE-conjugated anti-CD71 (BD Biosciences, 558040), and lineage cocktail of PE-Cy7-conjugated anti-CD45, Gr1 (eBioscience, 25-0451-82), and CD11b (Biolegend, 10126) antibodies for 30 min on ice. The erythroid population was identified using the gating strategy previously published.(9) The nucleated erythroid progenitors were differentiated from the enucleated erythrocytes (RBC, reticulocytes) cells using Hoechst-33342 (Sigma) nuclear staining. RBC and reticulocyte populations in peripheral blood were separated using PE-conjugated anti-CD71 (transferrin receptor) antibody. All samples were analyzed on FACSCanto flow cytometer (Becton-Dickinson Corporation, Mountainview, CA), and the analysis was conducted using the BD FACSDiva software (Becton-Dickinson Corporation, Mountainview, CA).

For ROS estimation, samples were washed with FACS buffer (1X PBS, 0.5% bovine serum albumin) after staining for cell surface markers, as described above, and then incubated in 100 µL of CM-H2DCFDA (Thermo Fisher C6827) at a 1:200 dilution for 30 min in a 37°C humidified incubator. CM-H2DCFDA fluorescence was analyzed on the FACSCanto using 488 nm excitation and 510nm emission wavelengths.

Tetramethylrhodamine, ethyl ester (TMRE), a fluorescent dye, was used to measure mitochondrial membrane potential, where healthy, functional mitochondria with more polarized membranes accumulated more dye (a robust fluorescent signal). After samples were stained for surface markers, they were washed and then incubated in 500 µL of TMRE (Life Technologies) at 0.01 µM for 30 min in a 37°C humidified incubator. For TMRE fluorescence staining, samples were analyzed on the FACS Canto using 488nm excitation and 580nm emission (PE channel).

Mitochondrial mass was estimated using an anti-TOM20 antibody (Abcam, ab186734), which labels the outer mitochondrial membrane.(10, 11) After staining cell surface markers, samples were washed and then fixed and permeabilized using BD Fix and Perm solution. Samples were then incubated with rabbit anti-TOM20 monoclonal antibody (Abcam, ab186734) in a 1:100 dilution at RT for 1 hour. Samples were then washed and incubated with FITC-conjugated donkey anti-rabbit IgG secondary antibody (Invitrogen) at a 1:100 dilution for 1 hour at RT. For nuclear staining, Hoechst (1:100 dilution) was added at the time of secondary antibody staining.

Imaging Flow Cytometry (IFC) analysis: Fixation and permeabilization of single-cell spleen suspension were performed as previously described. Cells were then incubated with APC-conjugated anti-Ter119 (Biolegend,116212), PE-conjugated anti-CD71 (BD Bioscience, 558040) as per the manufacturer's instructions, and then washed with phosphate-buffered saline, followed by incubation with rabbit anti-TOM20 monoclonal antibody (Abcam, ab186734) in a 1:100 dilution at RT for 1 hour. Samples were then washed and incubated with FITC-conjugated donkey anti-rabbit IgG secondary antibody (Invitrogen) at a 1:100 dilution for 1 hour at RT. For nuclear staining, Hoechst (1:100 dilution) was added at the time of secondary antibody staining. The distribution of mitochondria was studied using Amnis Imagestream^{X} analysis, and at least 10,000 events per sample were collected and analyzed with the associated Image Data Exploration and Analysis Software (IDEAS; Amnis) at 40 x/numerical aperture 0.75 and 60x/numerical aperture 0.9 objective lenses.

References

1. C. Paszty *et al.*, Transgenic knockout mice with exclusively human sickle hemoglobin and sickle cell disease. *Science* **278**, 876-878 (1997).
2. Y. Nakano *et al.*, Mutation of the Cyba gene encoding p22phox causes vestibular and immune defects in mice. *J Clin Invest* **118**, 1176-1185 (2008).
3. A. C. Heinrich, R. Pelanda, U. Klingmuller, A mouse model for visualization and conditional mutations in the erythroid lineage. *Blood* **104**, 659-666 (2004).
4. A. Hinge *et al.*, Asymmetrically Segregated Mitochondria Provide Cellular Memory of Hematopoietic Stem Cell Replicative History and Drive HSC Attrition. *Cell Stem Cell* **26**, 420-430 e426 (2020).
5. S. Roy *et al.*, Angiotensin receptor signaling in sickle cell anemia has a reno-protective effect on urine concentrating ability but results in sickle glomerulopathy. *Am J Hematol* **93**, E177-E181 (2018).
6. P. I. Arumugam *et al.*, Genetic diminution of circulating prothrombin ameliorates multiorgan pathologies in sickle cell disease mice. *Blood* **126**, 1844-1855 (2015).
7. M. S. Eiymo Mwa Mpollo *et al.*, Placenta growth factor augments airway hyperresponsiveness via leukotrienes and IL-13. *J Clin Invest* **126**, 571-584 (2016).
8. K. H. Chang *et al.*, Vasculopathy-associated hyperangiotensinemia mobilizes haematopoietic stem cells/progenitors through endothelial AT(2)R and cytoskeletal dysregulation. *Nat Commun* **6**, 5914 (2015).
9. J. Liu *et al.*, Quantitative analysis of murine terminal erythroid differentiation in vivo: novel method to study normal and disordered erythropoiesis. *Blood* **121**, e43-49 (2013).
10. G. L. Norddahl *et al.*, Accumulating mitochondrial DNA mutations drive premature hematopoietic aging phenotypes distinct from physiological stem cell aging. *Cell Stem Cell* **8**, 499-510 (2011).
11. M. J. de Almeida, L. L. Luchsinger, D. J. Corrigan, L. J. Williams, H. W. Snoeck, Dye-Independent Methods Reveal Elevated Mitochondrial Mass in Hematopoietic Stem Cells. *Cell Stem Cell* **21**, 725-729 e724 (2017).
12. T. Brattelid *et al.*, Expression of mRNA encoding G protein-coupled receptors involved in congestive heart failure--a quantitative RT-PCR study and the question of normalisation. *Basic Res Cardiol* **102**, 198-208 (2007).

Figure S1

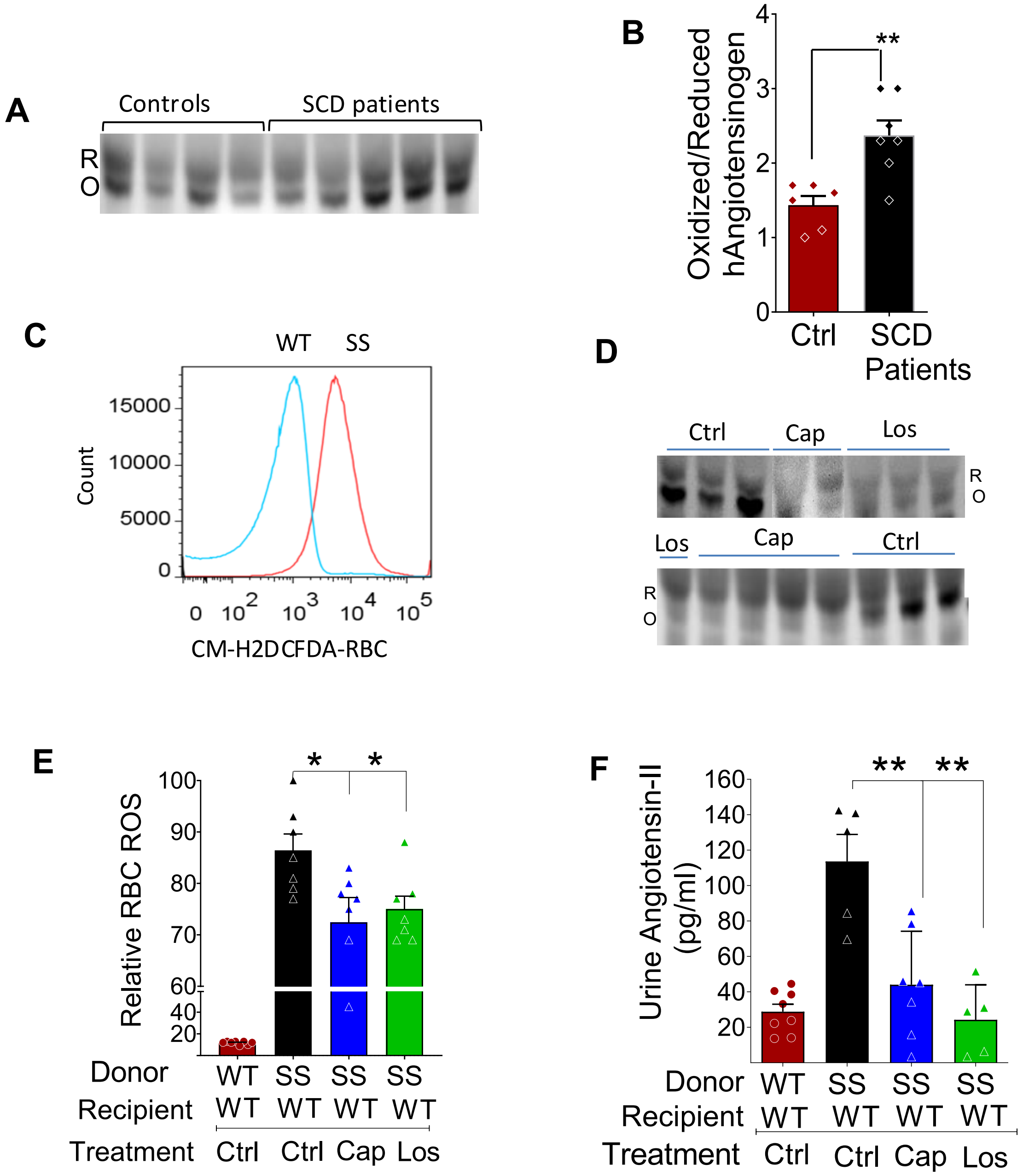

Supplemental Figure S1. ROS-RAS axis in sickle cell disease (A-B) Representative western blot analysis of oxidized and reduced angiotensinogen in plasma (**A**), and quantification of the angiotensinogen bands from the western blots **(B)** are shown in sickle cell disease (SCD) patients and healthy sibling controls. Each lane represents an individual patient. Average data is from n= 6 individuals/group. (**C**) Representative histogram of CM-H_2-DCFDA (ROS) staining of RBC in Berkeley SCA (SS) and wild-type (WT) mice. (**D**) Representative Western blot of reduced and oxidized angiotensinogen in SS mice treated with captopril, losartan and vehicle (Control n= 6, Captopril n=6, Losartan n= 4) (**E**) RBC ROS levels in peripheral blood and **(F)** Urine angiotensin-II levels in WT mice transplanted with SCA hematopoiesis (SS/WT [donor/recipient]) that were fully chimeric for donor, placed on captopril (Cap; blue bar) or losartan (Los; green bar) or treated with vehicle control (Ctrl; black bar) versus those transplanted with WT hematopoiesis (CD45.2+) into WT (CD45.1+) controls (Ctrl; red bar) (n=5-8 mice/group). Unpaired t-tests were used to analyze panel B and ANOVA for panels E-F. Statistical significance is denoted by *P<0.05, **P<0.01, ***P<0.001, ****P<0.0001, ns=not significant.

Figure S2

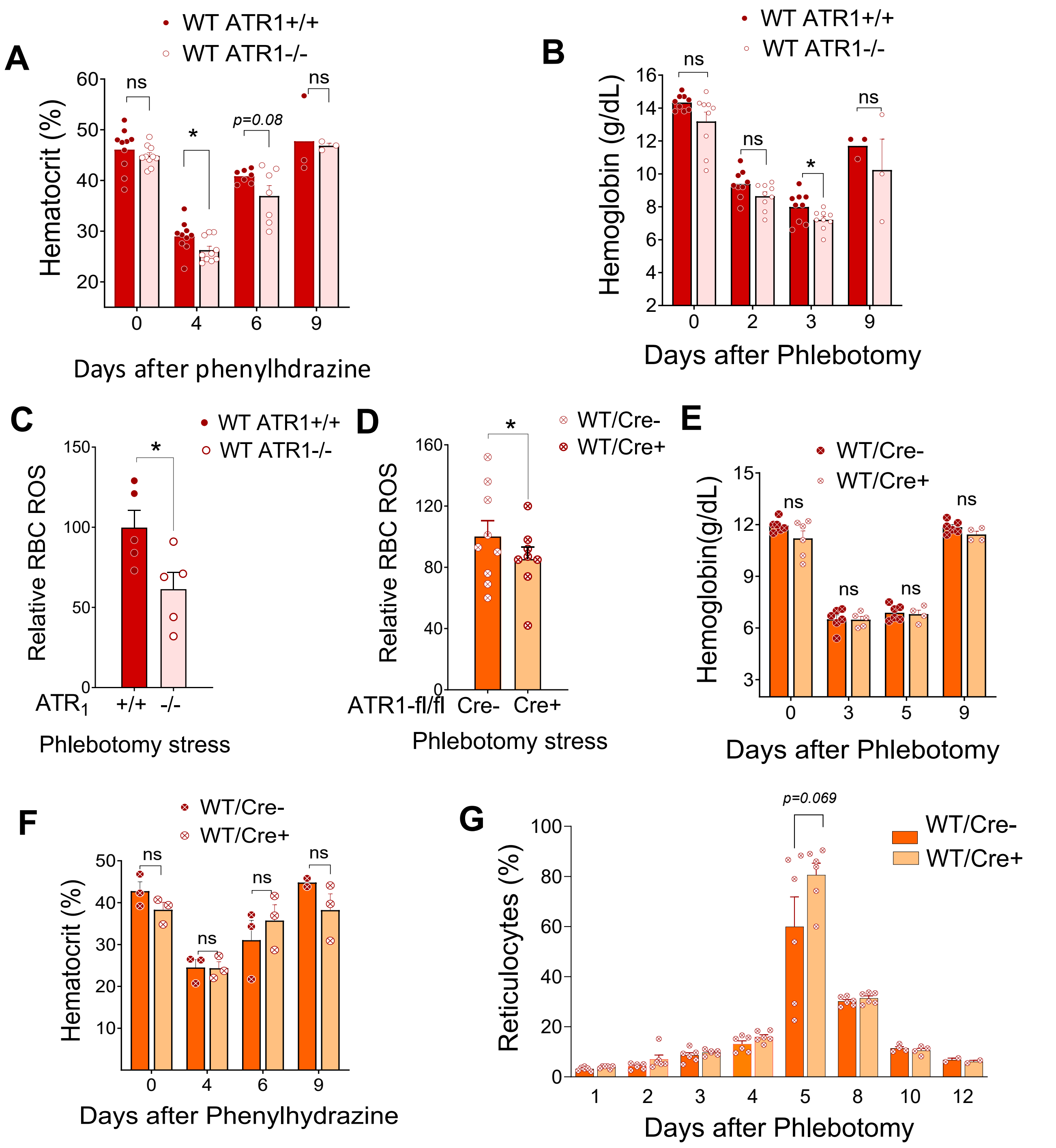

Supplemental Figure S2. Stress Erythropoiesis in WT mice with germline deficiency (ATR1$^{-/-}$) and erythroid-specific deficiency of ATR1 (ATR1$^{fl/fl}$ EpoR Cre+; [Cre+]). WT ATR1$^{-/-}$ were more anemic at the peak of erythropoietic stress (Day 3-4) induced by (**A**) phenylhydrazine and (**B**) phlebotomy compared to the WT (ATR1$^{+/+}$) control mice. (**C-D**) Cumulative data on the relative CM-H_2-DCFDA mean fluorescence intensities in RBC in phlebotomy-stressed mice with germline ATR1 deficiency (**C**), and erythroid-specific ATR1-deficient mice (WT ATR fl/fl Cre+; labelled as WT/Cre+) (**D**), when compared to their respective stressed ATR1$^{+/+}$ and WT ATR fl/fl Cre- (labelled as WT/Cre-) controls. (**E**) Hemoglobin and (**F**) Hematocrit levels in WT/Cre+ and WT/Cre- controls at the peak of erythropoietic stress (n=3-10 mice/group; WT ATR1$^{+/+}$ dark red bar, WT ATR1$^{-/-}$ light red bar, WT/Cre- dark orange, WT/Cre+ light orange). (**G**) The compensatory peak reticulocyte response to the erythropoietic stress in WT/Cre+ trended higher than WT/Cre- mice (n=2-6 mice/group/time-point). Statistical analysis was done comparing ATR1-deficient and control mice using t-test or Mann-Whitney test. Statistical significance is denoted by * P<0.05. ns= not statistically significant.

Figure S3

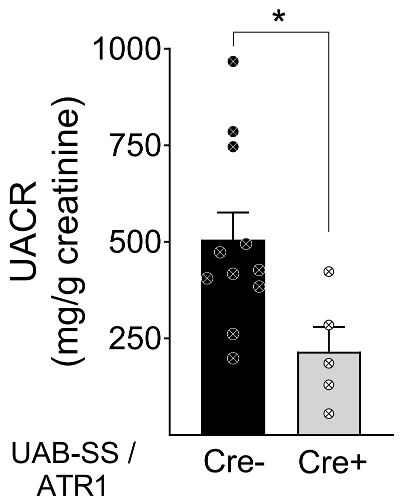

Supplemental Figure S3. UAB-SS mice with erythroid-specific deficiency of ATR1 (UAB-SS/Cre+; grey bar) had lower UACR (urine albumin creatinine ratio) when compared to control UAB-SS mice (UAB-SS/Cre-; black bar). UAB-SS/Cre- n=11, UAB-SS/Cre+ n=5. Statistical analysis was done using Mann-Whitney test. Statistical significance is denoted by * P<0.05

Figure S4

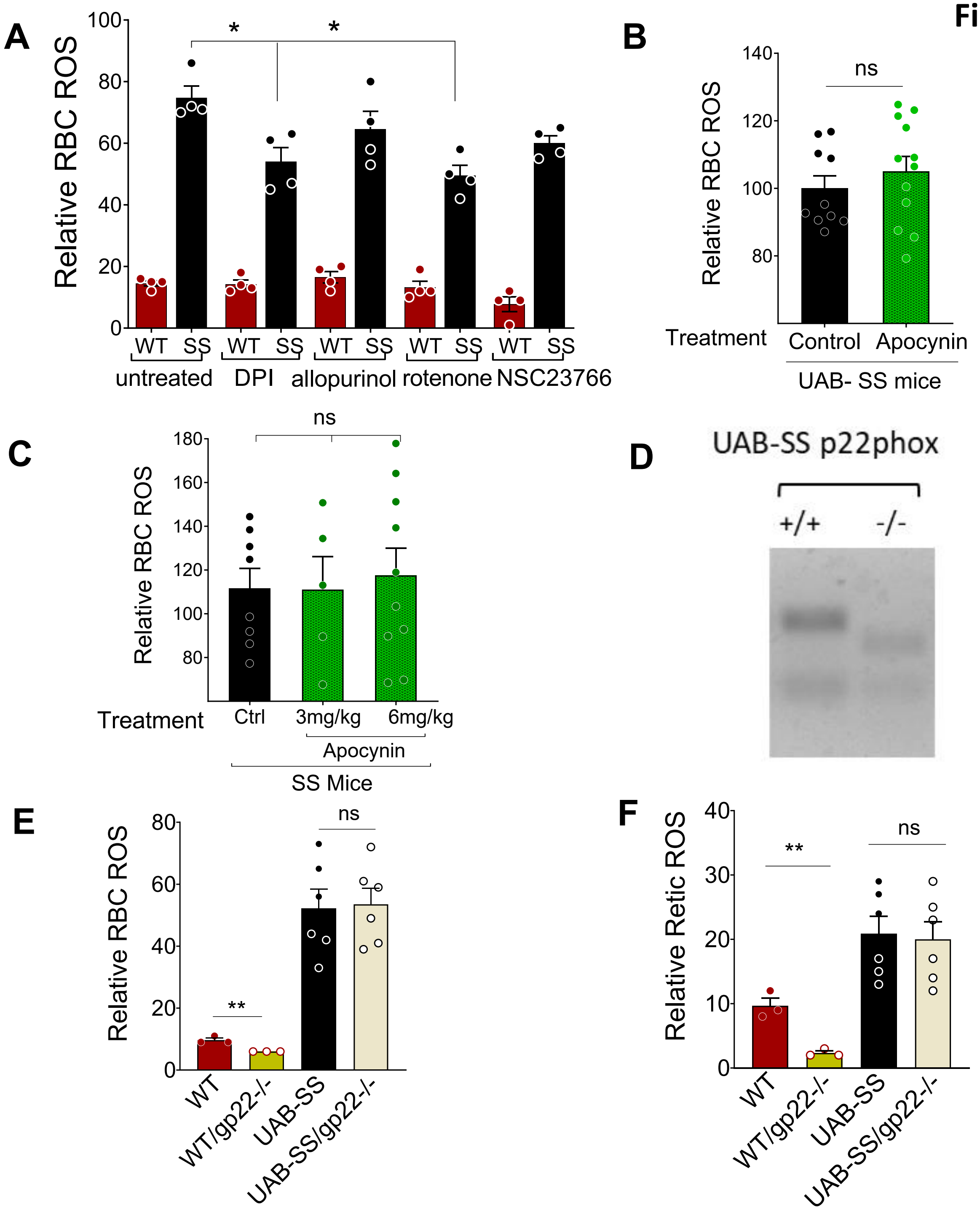

Supplemental Figure S4. The cumulative data on the relative CM-H_2-DCFDA mean fluorescence intensities in RBC *in vitro* (relative change in mean RBC ROS compared to the WT mean RBC ROS) in **(A)** WT (solid red bar) and Berkeley SCA (SS) (solid black bar) mouse RBC following incubation with the following pharmacological inhibitors of ROS: diphenyleneiodonium chloride (DPI; 50µM), a broad inhibitor of NADPH oxidases, allopurinol (500µM), a xanthine oxidase inhibitor, rotenone (50µM), a mitochondrial electron transport inhibitor, and NSC23766 (500µM) a Rac GTPase 1-3 inhibitor. (n=4 mice/group). (**B-C**) Relative RBC ROS from peripheral blood UAB-SS (**B**) and SS RBC (**C**) following selective pharmacological inhibition of NADPH oxidases with apocynin (shaded green bar) for 24 weeks (n= 5-11 mice/group). (**D**) Representative results of PCR (on somatic DNA) show bands for p22phox+/+ (89bp & 202bp) and p22phox-/- (89bp, 162bp) genotype mice (**E**) Relative RBC ROS and (**F**) Relative reticulocyte ROS levels in peripheral blood obtained from WT and UAB-SS mice with or without deficiency of p22phox; (n = 3-6 mice/group). Statistical analysis was done using unpaired t-tests with data shown as mean ± SEM. Statistical significance is denoted by * P<0.05, **P<0.01, ns = not significant.

Figure S5

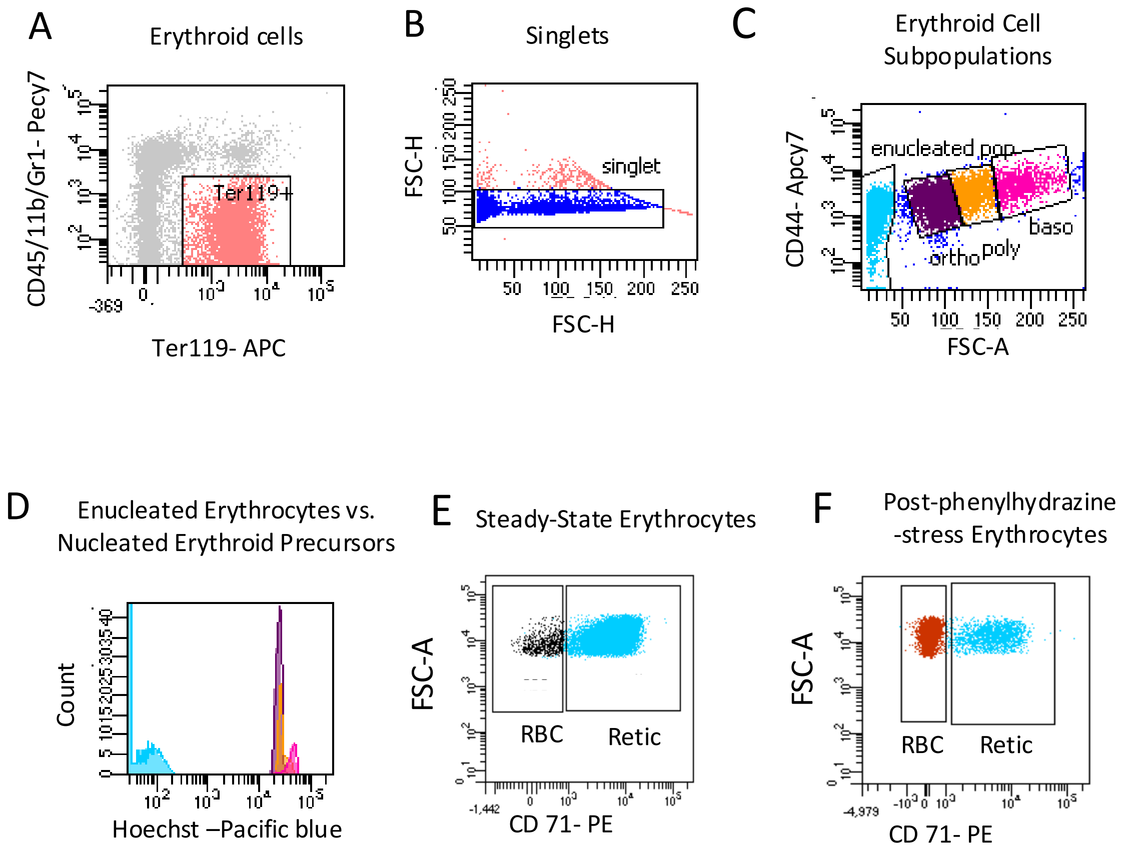

Supplemental Figure S5. Representative flow cytometry dot plots showing the gating strategy to identify the erythroid sub-populations. (A) Erythroid cells that stained for Ter119 and were negative for CD45/CD11b/Gr1 were gated, and **(B)** single cells (singlets) within this erythroid population were further analyzed, **(C)** based on size (FSC) and the level of CD44 expression to identify the nucleated erythroid precursor subpopulations and enucleated erythrocytes. The nucleated erythroid precursor populations were gated as basophils (baso), polychromatophils (poly), orthochromatophils (ortho), and enucleated erythrocytes consisted of both reticulocyte and RBC. **(D)** The enucleated erythrocytes (reticulocytes and RBC) were further confirmed by Hoechst staining, showing they did not stain for this nuclear stain and confirmed correct gating of this population in panel (C). **(E, F)** Within the erythrocyte Hoechst- population, reticulocytes (retic) were separated from mature RBC by CD71 staining. Gating for the separation of reticulocytes and RBC in the erythrocyte populations at steady-state and post-phenylhydrazine stress are shown in panels E and F, respectively

Figure S6

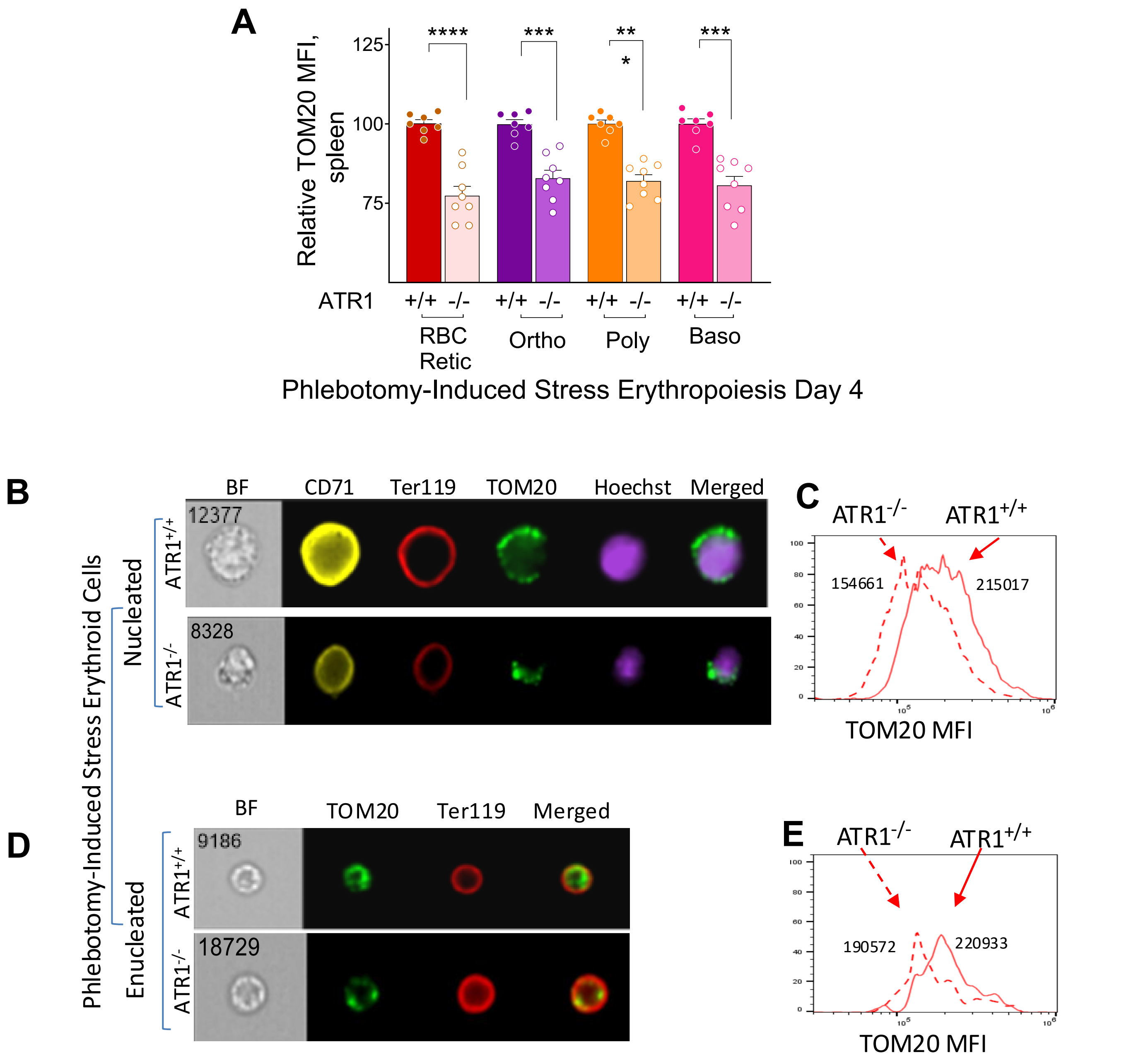

Supplemental Figure S6. AT signaling deficiency reduces mitochondrial mass in stressed erythroid cells and stressed erythrocytes. (A) Cumulative data on the mean fluorescence intensities of TOM20 labeled erythroid subpopulations (reflecting mitochondrial mass) acquired at the peak of phlebotomy-induced stress in ATR1$^{-/-}$ mice (light shaded bars) vs. control ATR1$^{+/+}$ mice (dark-shaded bars); red-RBC/retic, purple- orthochromatic, yellow- polychromatophilic, pink- basophilic compartments. (n=7-8 mice in each group). Statistical analysis was performed using multiple t-tests to compare the specific erythroid subpopulations. Statistical significance is denoted by * $P<0.05$, **$P<0.01$, ***$P<0.001$, ****$P<0.0001$. (**B- E**) Representative figures from Amnis image stream analysis done on phlebotomy stressed ATR1$^{-/-}$ mice (mitochondria labeled with Tom20) (**B**) nucleated erythroid precursors (CD71+, Ter119+, Hoechst+) and (**D**) enucleated erythrocytes (CD71-, Ter 119+, Hoechst-) compared to control stressed ATR1$^{+/+}$ mice. The number shown on the top left inside the Bright-field (BF) images in panels **B** and **D** is the unique identification number of that particular cell. The TOM20 mean fluorescence intensities (MFIs) are shown in panels C and E and depicted as dashed histograms for ATR1$^{-/-}$ and solid histograms for ATR1$^{+/+}$.

Figure S7

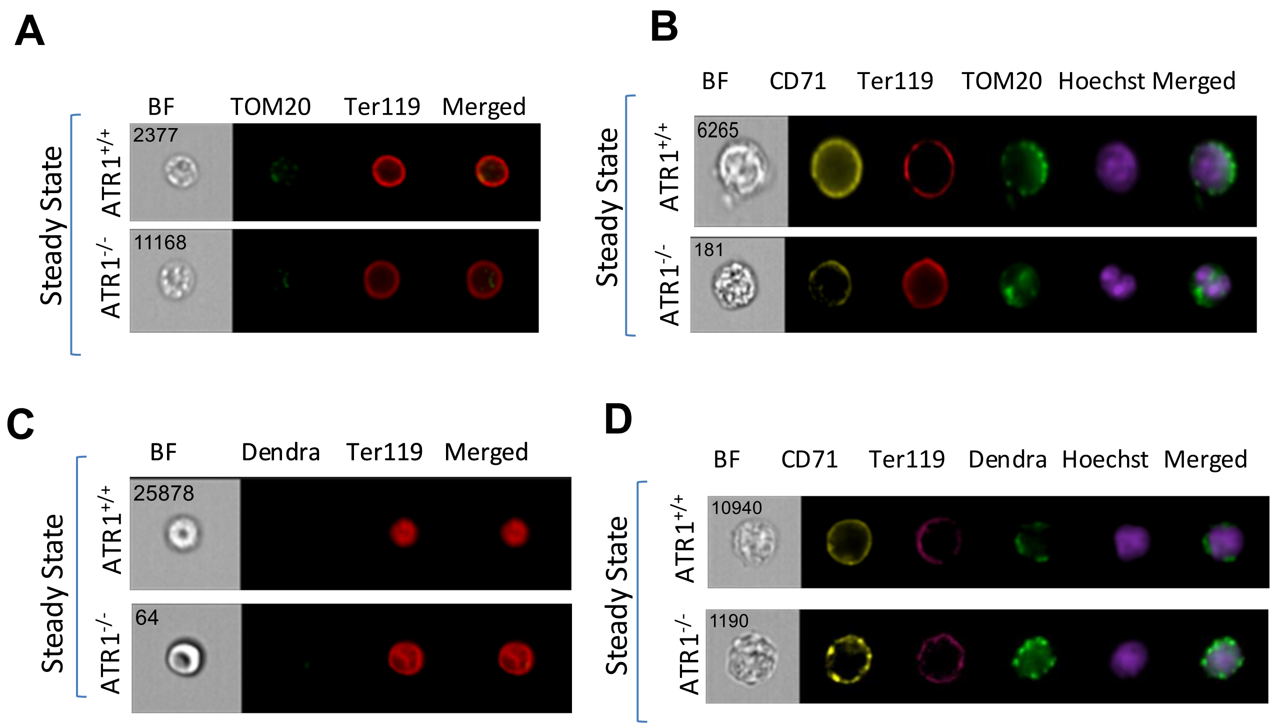

Supplemental Figure S7. Deficiency of AT signaling does not affect erythroid cell and erythrocyte mitochondrial mass in unstressed (baseline) erythropoiesis. (**A, B**) Representative images from Amnis Image-Stream analysis of erythrocytes (A) and nucleated erythroid cells (B) from ATR1$^{-/-}$ and ATR1$^{+/+}$ mice at steady-state labeled with Ter119, TOM20, Hoechst, and CD71. (**C, D**) Representative images from Amnis Image-Stream analysis of erythrocytes (C) and nucleated erythroid cells (D) from Mitodendra2 ATR1$^{-/-}$ and Mitodendra2 ATR1$^{+/+}$ mice at steady state. The number shown on the top left inside the Bright-field (BF) images in panels A-D is the unique identification number of that particular cell

Figure S8

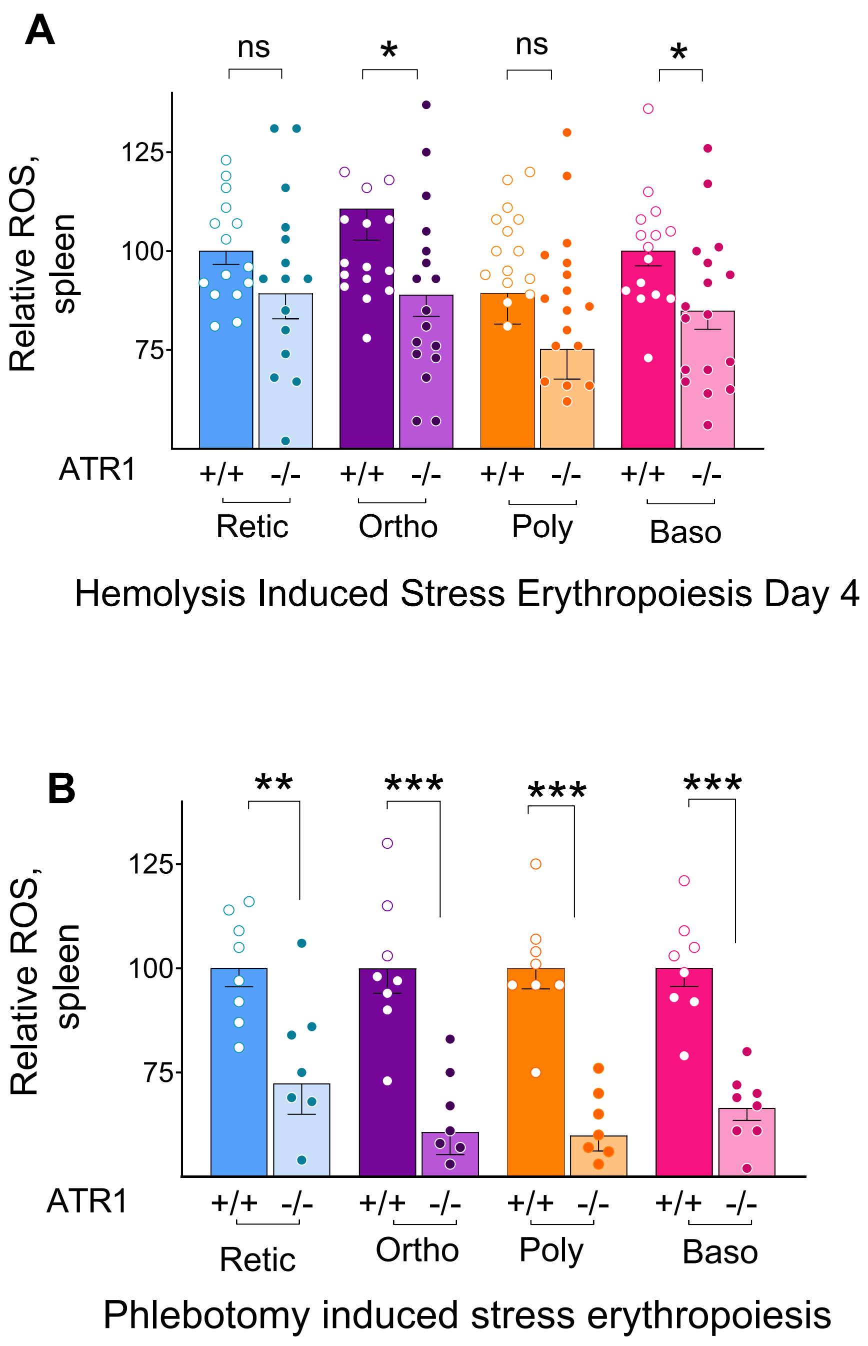

Supplemental Figure S8. Deficiency of AT signaling in stressed erythroid cells decreases levels of reactive oxygen species (ROS) ROS levels (relative CM-H_2-DCFDA MFI) in erythroid compartments obtained from spleens of WT ATR1$^{+/+}$ and ATR1$^{-/-}$ mice **(A)** stressed with phenylhdrazine (n= 15 mice/group), and (**B**) stressed with daily phlebotomy (n= 8 mice/group). Statistical analysis was done using Mann-Whitney test. Statistical significance is denoted by * P<0.05, **P<0.01, ***P<0.001, ****P<0.0001

Figure S9

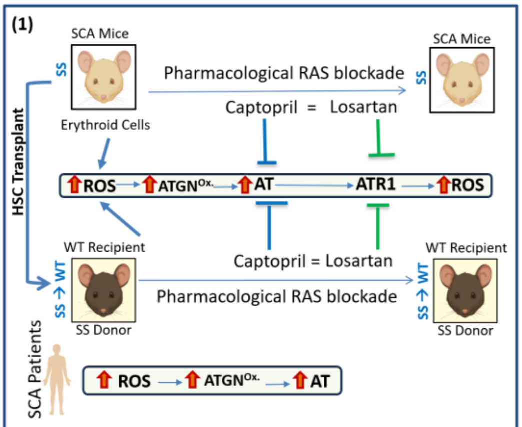

Experimental Schema for Fig 1 and S1 to demonstrate that AT→ATR1→ROS Axis is Mediated by SCA Erythroid Cells. (i) Mechanism underlying hyperangiotensinemia in SCA is an increased ROS-RAS feedback loop, driven by ATR1 expressed on erythroid cells. (ii) Pharmacological blockade of AT or ATR1 interrupts this loop, reducing ROS and AT. (iii) Transplant of SCA hematopoiesis into WT mice recreates the increased ROS-RAS feedback loop, again interrupted by pharmacological AT or ATR1 blockade. (iv) The effect of AT blockade is similar to that of ATR1 blockade, indicating that it occurs via AT→ATR1 signaling. (v) A similar ROS-RAS axis is seen in human SCA patients.

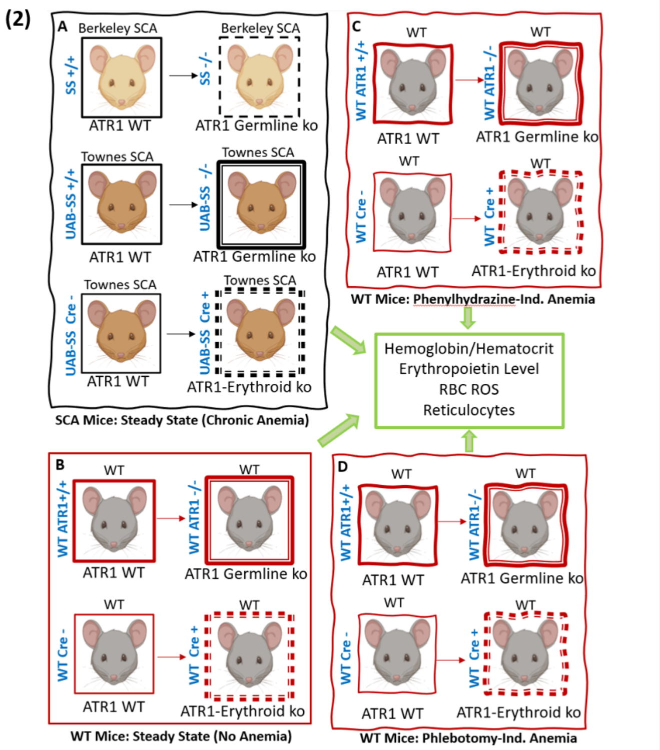

Experimental Schema for Fig. 2 and Fig. S2 to demonstrate that I. AT→ATR1 Signaling is Essential for Stress Erythropoiesis Not Steady-State Erythropoiesis. Hemoglobin/hematocrit, erythropoietin levels, and RBC ROS were assessed in: **(A)** SCA [Berkeley (SS), Townes (UAB-SS)] mice with and without germline, and with and without erythroid-specific deficiency of ATR1. **(B)** WT (C57Bl/6) mice with germline deficiency and without erythroid-specific deficiency at steady-state/baseline. Experimentally inducing stress erythropoiesis/anemia in WT mice with **(C)** phenylhydrazine administration and **(D)** daily phlebotomies for 3 consecutive days.

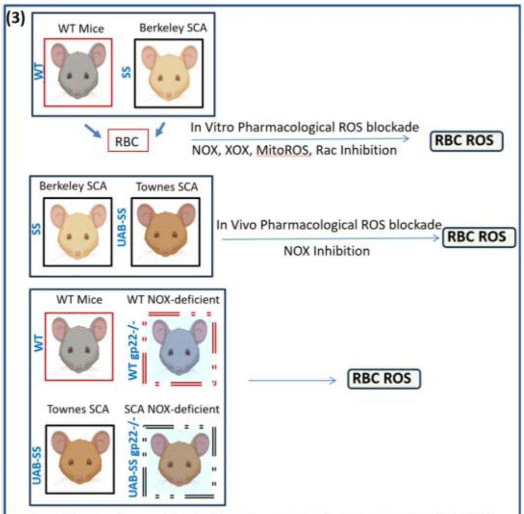

Experimental Schema for Fig S4 to demonstrate that the canonical AT→NOX signaling does not affect RBC ROS in stress erythropoiesis. RBC ROS was measured after Pharmacological inhibition of WT and SCA (SS) RBCs in vitro with inhibitors of NADPH oxidase (NOX) and mitochondrial electron transport. In vivo administration of a NOX antagonist to SS or UAB-SS mice. WT and UAB-SS mice with genetic knockout of gp22, which is the common subunit of all NOX isoforms.

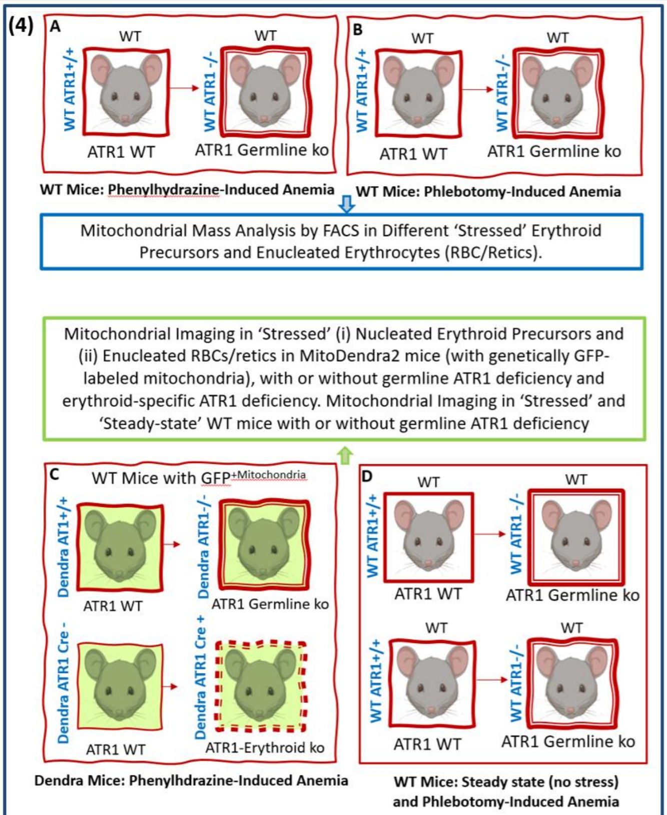

Experimental Schema for Fig. 3 and Figs. S5-7 to determine the mitochondrial mass by flow cytometry and Image Stream Analysis in WT mice(with and without ATR1 germline deficiency) and Dendra mice (with and without ATR1 germline or erythroid-specific deficiency). In WT mice, Tomm20 was used to stain for mitochondrial mass

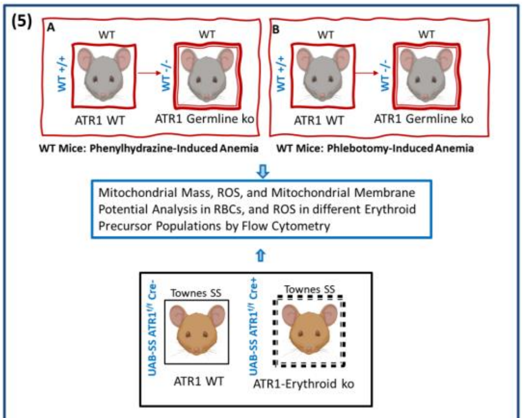

Experimental Schema for Fig. 4 and Fig. S8 to demonstrate that increased AT→ATR1 Signaling due to stress erythropoiesis results in higher retention of dysfunctional mitochondria with increased ROS in erythroid precursors and RBC in: **(A)** WT (C57Bl/6) mice with and without germline deficiency of ATR1 subjected to phenylhydrazine-induced stress erythropoiesis. **(B)** WT mice with and without germline deficiency of ATR1 with phlebotomy-induced stress erythropoiesis. **(C)** UAB-SS (Townes SCA) mice with and without erythroid-specific deficiency of ATR1
