# Supplementary material for: Angiotensin signaling is essential for stress erythropoiesis but causes retention of dysfunctional mitochondria in RBCs
Source: JCI Insight. 2026 Mar 12;11(9):e200722. doi: 10.1172/jci.insight.200722 (PMC13232005; doi:10.1172/jci.insight.200722)

# Unedited gels for figures

# Total Angiotensinogen – Western Blots

# Figure 1A:

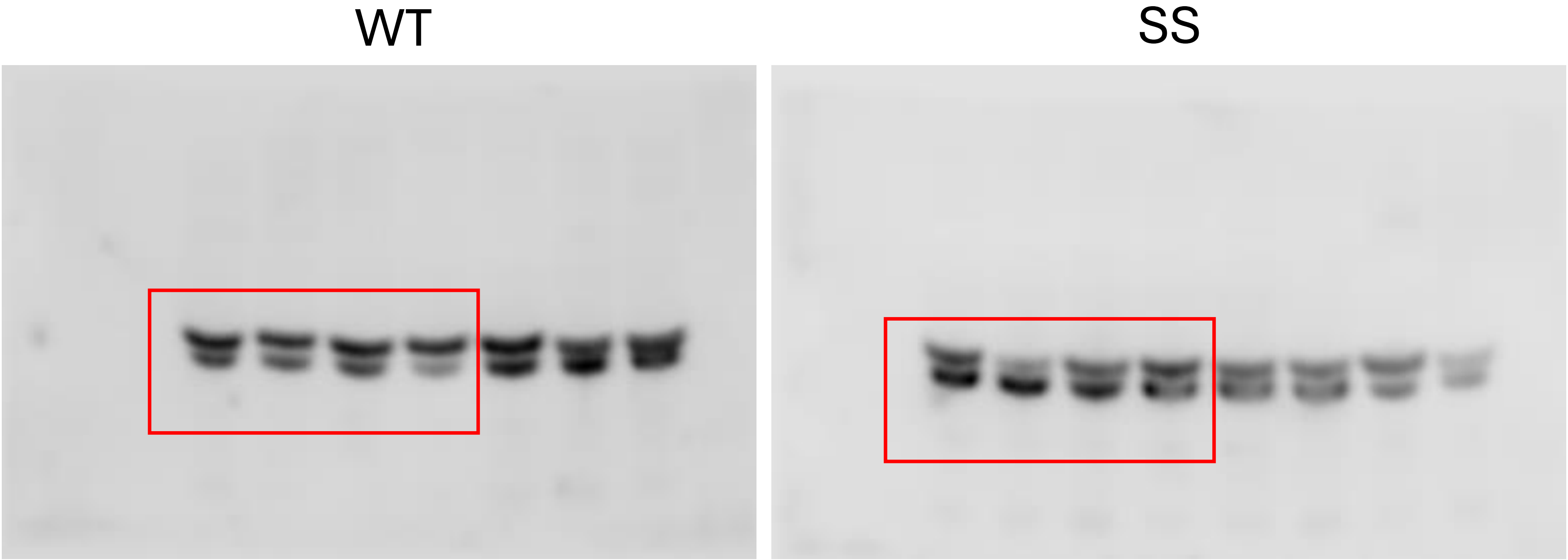

# Angiotensinogen-Redox Western Blot
# Figure 1B (AA)

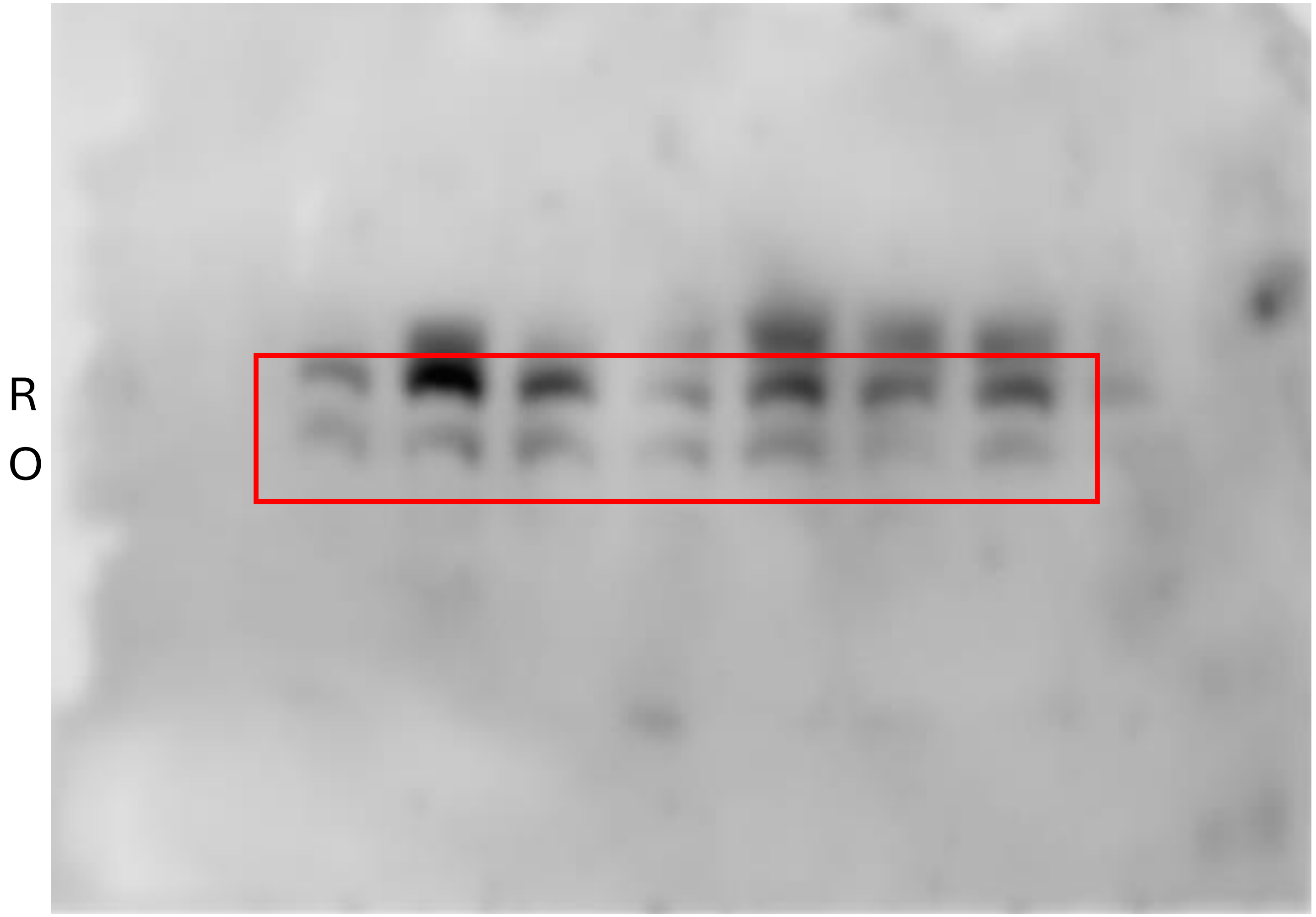


** The lanes inside the red box have been shown in the manuscript figure

# Angiotensinogen-Redox Western Blot Figure-1B (SS)

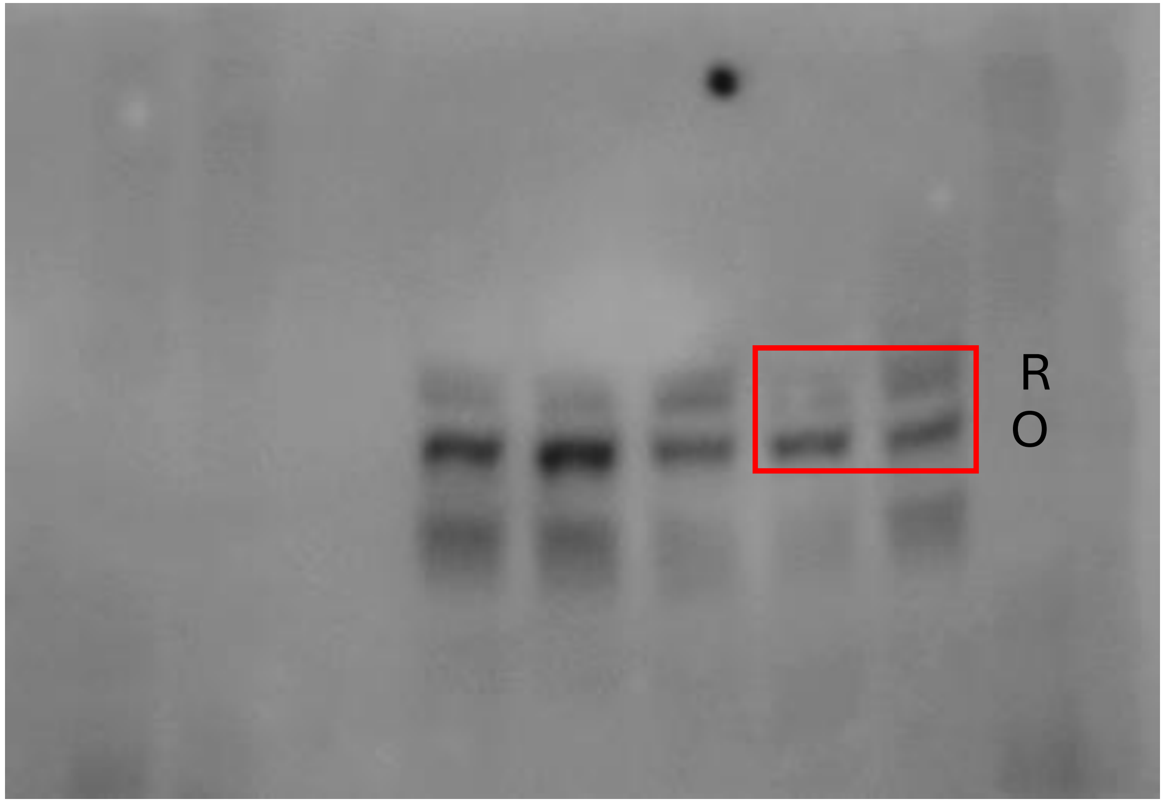


The lanes inside the red box have been shown in the manuscript figure

# Angiotensinogen-Redox Western Blot
# Figure-1B (SS)

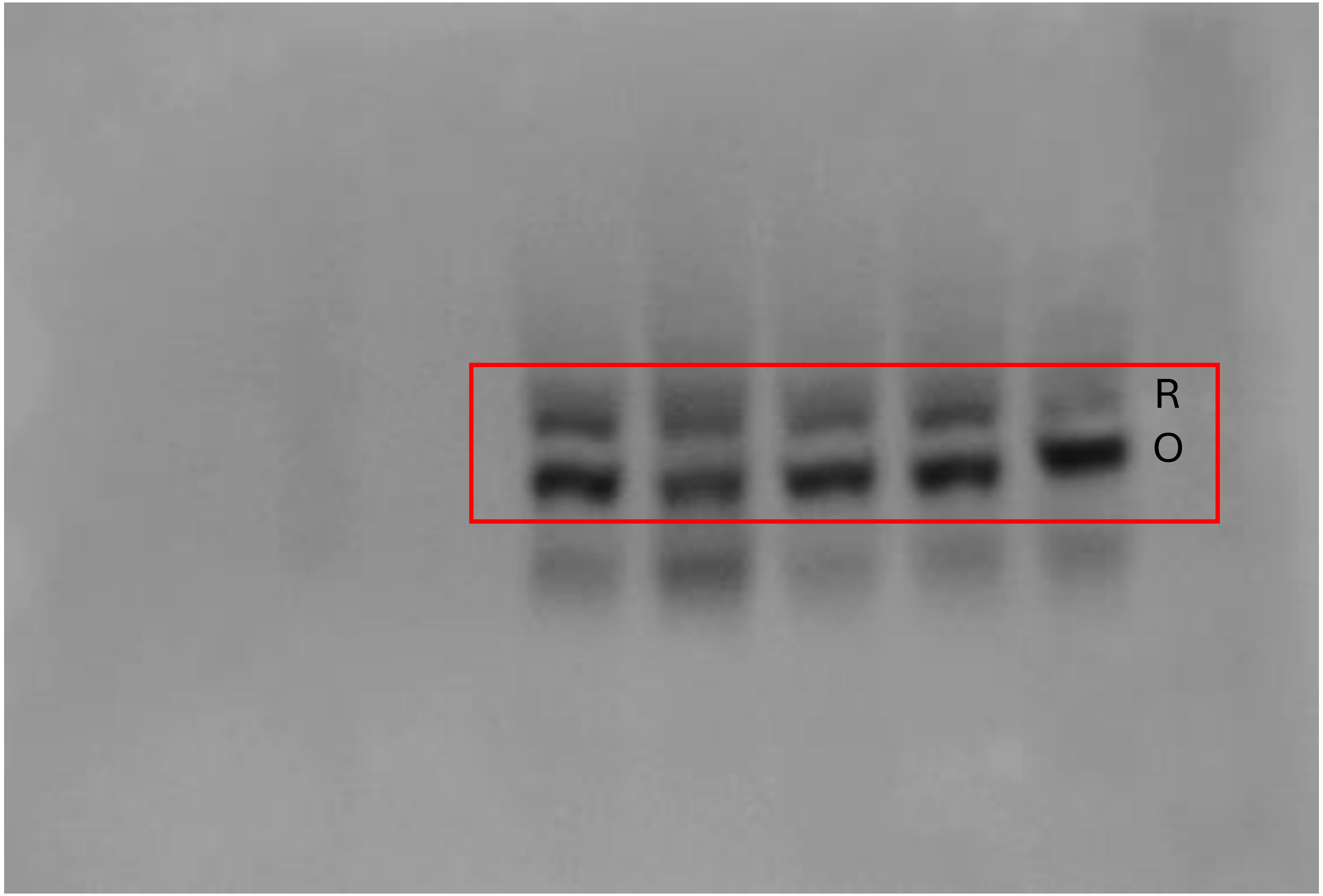

# Angiotensinogen-Redox Western Blot Figure S1A

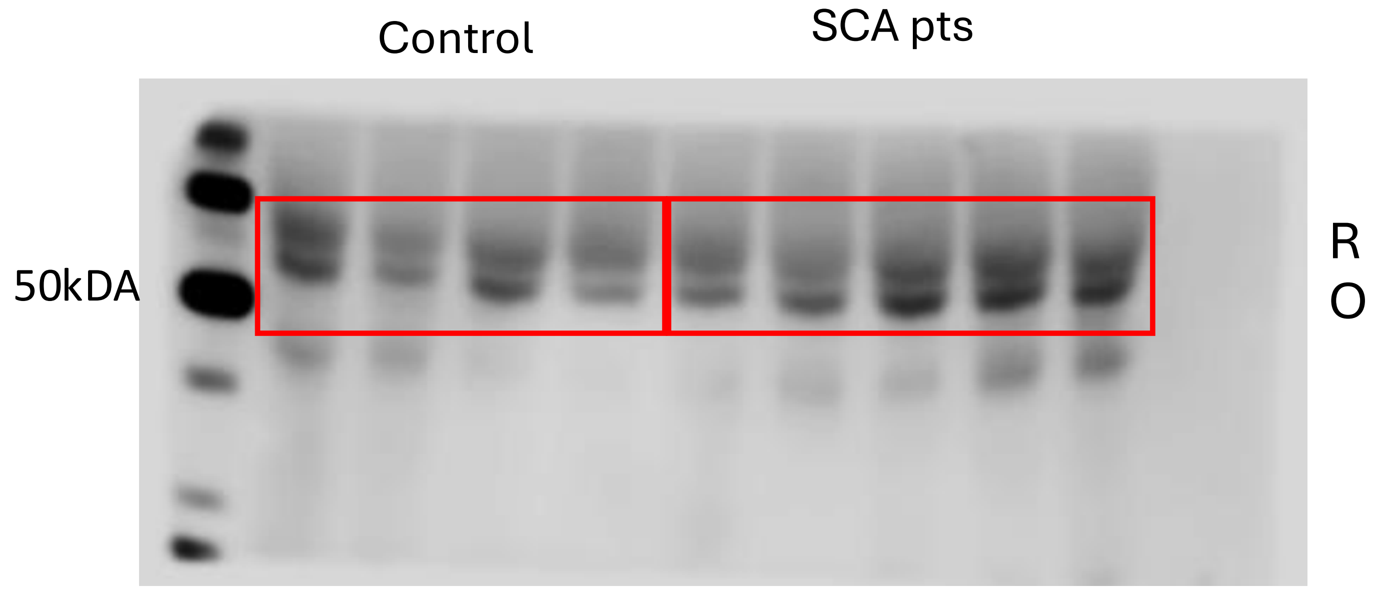

# Angiotensinogen-Redox Western Blot
# Figure S1D

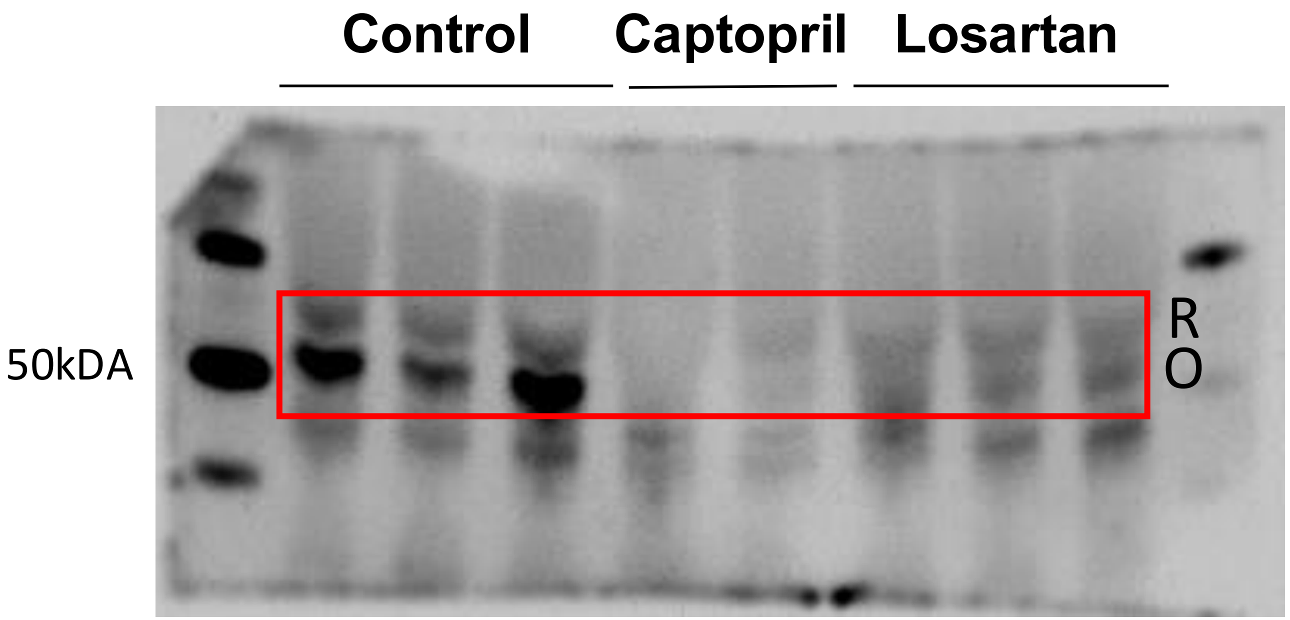

# Angiotensinogen-Redox Western Blot
# Figure S1D

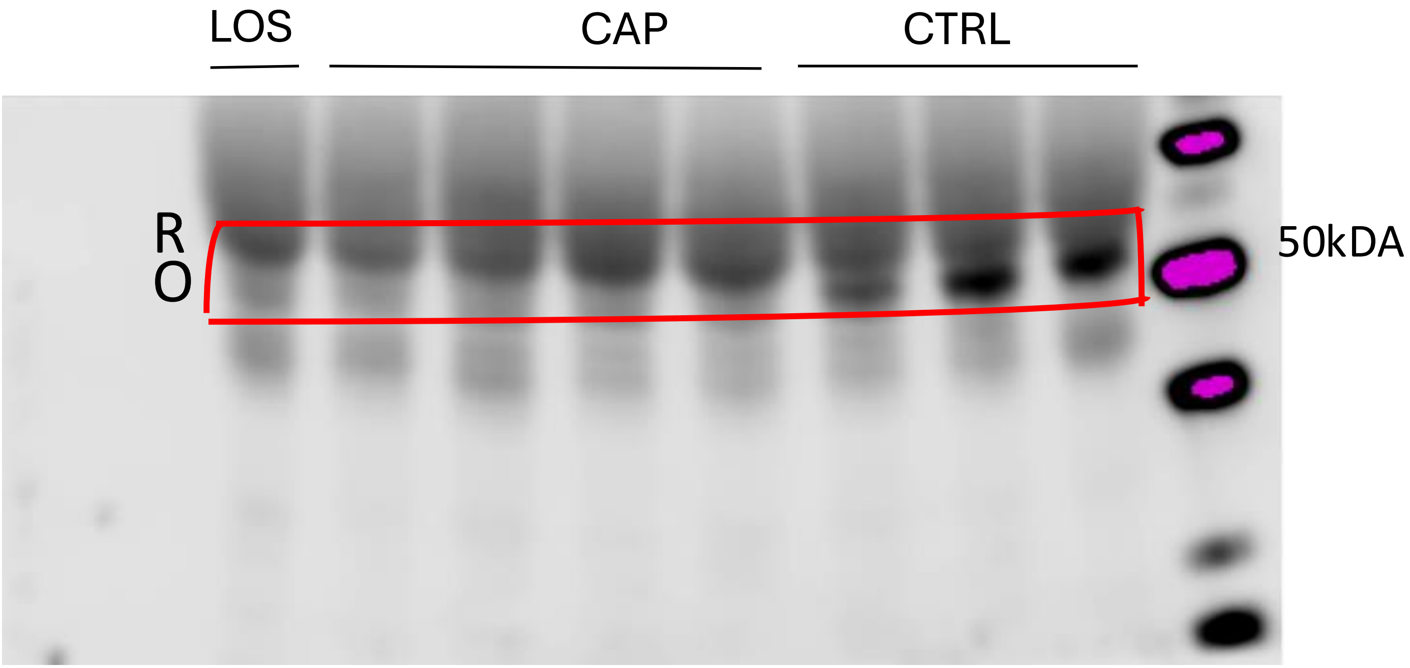

Supplement: Unedited blot and gel images [file jciinsight-11-200722-s059.pdf]
